# Investigating cross-lingual training for offensive language detection

**DOI:** 10.7717/peerj-cs.559

**Published:** 2021-06-25

**Authors:** Andraž Pelicon, Ravi Shekhar, Blaž Škrlj, Matthew Purver, Senja Pollak

**Affiliations:** 1 Jožef Stefan Institute, Ljubljana, Slovenia; 2 Jožef Stefan International Postgraduate School, Ljubljana, Slovenia; 3 Queen Mary University of London, London, United Kingdom

**Keywords:** Cross-lingual models, Transfer learning, Intermediate training, Offensive language detection, Deep learning

## Abstract

Platforms that feature user-generated content (social media, online forums, newspaper comment sections etc.) have to detect and filter offensive speech within large, fast-changing datasets. While many automatic methods have been proposed and achieve good accuracies, most of these focus on the English language, and are hard to apply directly to languages in which few labeled datasets exist. Recent work has therefore investigated the use of *cross-lingual transfer learning* to solve this problem, training a model in a well-resourced language and transferring to a less-resourced target language; but performance has so far been significantly less impressive. In this paper, we investigate the reasons for this performance drop, via a systematic comparison of pre-trained models and intermediate training regimes on five different languages. We show that using a better pre-trained language model results in a large gain in overall performance and in zero-shot transfer, and that intermediate training on other languages is effective when little target-language data is available. We then use multiple analyses of classifier confidence and language model vocabulary to shed light on exactly where these gains come from and gain insight into the sources of the most typical mistakes.

## Introduction

The massive growth of social media in the last two decades has changed the way we communicate with each other. On the one hand, it allows people worldwide to connect and share knowledge; but on the other, there is a corresponding increase in the negativity to which they can be exposed. Offensive language and hate speech are major concerns on social media, and result in poor psychological well-being, hate crime, and minority group prejudice in both virtual and local communities (Blair, 2019; Gagliardone et al., 2015). As an extreme example, social media posts were one reason to incite violence against Rohingya Muslims in Myanmar in 2017 (Beyrer & Kamarulzaman, 2017; Stevenson, 2018; Subedar, 2018).

There is therefore a growing need to moderate these platforms to minimize hate speech. Platforms like Facebook, Twitter, and YouTube have started taking the necessary steps to monitor their platforms using manual moderation and automated detection (Simonite, 2020; Lomas, 2015). At the same time, countries like Germany (Lomas, 2017) and the UK (Morgan, 2020) are creating regulations to hold social media platforms accountable. However, with billions of messages posted daily on social media platforms, it is nearly impossible to do this manually, and automatic methods are becoming important. Multiple datasets (e.g., Davidson et al., 2017; Zampieri et al., 2019a; Ljubešić, Fišer & Erjavec, 2019), shared tasks (e.g., Wiegand, Siegel & Ruppenhofer, 2018; Zampieri et al., 2020a) and models (e.g., Salminen et al., 2018; Farha & Magdy, 2020; Gao & Huang, 2017; Zampieri et al., 2020a) have been proposed for several languages. However, so far, good accuracy in automatic detection depends upon the availability of substantial, well-labelled datasets: in many domains and in many languages, this is not the case.

A common theme across recent work in NLP which promises to reduce the requirement for such task-specific labeled data is the use of *transfer learning* (see e.g., Ruder, 2019). Typically, in this approach, a large pre-trained language model (LM) is learned using a general *source* task (e.g., masked language modeling or next sentence prediction) over a very large amount of easily obtained unlabeled data. This pre-trained LM—which contains a lot of information about word meaning and dependencies—can then be fine-tuned on the *target* NLP task (e.g., hate speech detection, question answering etc.), requiring only a smaller labeled dataset to achieve state-of-the-art performance (see e.g., Devlin et al., 2019).

While most of this research is focused on the English language only, the principle extends to transfer between languages, and recent work in *cross-lingual transfer* leverages datasets in multiple languages to provide pre-trained models with multilingual embeddings (Artetxe & Schwenk, 2019; Devlin et al., 2019). For example, Devlin et al. (2019) propose a multilingual version of BERT, called mBERT, trained on 104 languages, in which the representations seem to capture significant syntactic and semantic information across languages (Pires, Schlinger & Garrette, 2019). These pre-trained LMs can therefore be trained on a language with available resources and employed on a less-resourced target language without additional language-specific training. This can help alleviate the data availability gap between high-resourced and less-resourced languages: for example, Leite et al. (2020) perform zero-shot transfer from English to Brazilian Portuguese for toxic comment detection. Most such studies are however restricted to evaluating zero-shot transfer from one language to one other only, and using only one multilingual pre-trained LM. Furthermore, several studies (Stappen, Brunn & Schuller, 2020; Leite et al., 2020), including our own initial work (Pelicon et al., 2021), suggest that this *zero-transfer* approach to multilingual training does not achieve performance comparable to systems trained on the actual target language data. As such, some amount of data in the target language is still preferred and may be needed for good accuracy. However, it is not clearly understood how exactly the amount of data affects this requirement and the performance of the final models.

Another question that remains largely unexplored is whether this data shortage problem can instead be addressed by using training data in one or several other non-target languages. An *intermediate training* mechanism has been proposed (Yogatama et al., 2019; Wang et al., 2019a; Pruksachatkun et al., 2020; Vu et al., 2020) to reduce the need for large scale data for all tasks in all languages. In the intermediate training step, instead of fine-tuning the LM directly on the target language task, it is first trained on a similar task using the same or different language data. Pruksachatkun et al. (2020) show that performing intermediate training using English data improves the multiple XTREME benchmark tasks (Hu et al., 2020). Robnik-Sikonja, Reba & Mozetic (2020) perform sentiment classification using training data from both target language and several non-target languages. However, this work is evaluated only in a setting where all available target language data is used for training: it is therefore hard to tell whether and how the benefit of intermediate training depends on how much target data is available. Stappen, Brunn & Schuller (2020) investigate this, via an analysis of cross-lingual capabilities of their hate speech model in which they first train a model in one language and then progressively add data in the target language. However, their analysis is performed only on one pair of languages. From these studies alone it is therefore not yet clear how much of the performance gap is due to the pre-trained model and its properties, and how much to the training regime, choice of intermediate languages and relative amount of data available.

In this work we perform a thorough analysis of the feasibility of training models that leverage multilingual representations with non-target language data. Specifically, we address the following research questions:

- *Effect of pre-trained LM:* How does the choice of multilingual pre-trained language model affect performance?
- *Effect of intermediate training:* Where little or no target language training data is available, when and by how much does intermediate training in a different language boost performance?
- *Data hunger of the model:* How does performance depend on the amount of intermediate and/or target language data?

We used five hate speech datasets in different languages, namely Arabic, Croatian, German, English, and Slovenian. All these languages are included in the standard pre-trained mBERT model. Arabic, German and English were chosen for their range of similarity: while German is fairly similar to English, sharing many syntactic and vocabulary features, Arabic is dissimilar to both, with very different linguistic features, an entirely different alphabet, and written right-to-left rather than left-to-right. Croatian and Slovenian were then chosen for being less-resourced, for representing a mid-point in similarity (being Slavic languages, they are less similar to English than German is, but more so than Arabic), and because they are included in a more specific trilingual Croatian-Slovenian-English pre-trained language model based on BERT architecture (Ulčar & Robnik-Šikonja, 2020, see “Background and Related Work”). This selection allows us to test a range of hypotheses, including that intermediate training may be more useful for more similar languages and that more specific LMs transfer better. We show that cross-lingual transfer can be useful for the offensive language detection task, and that using a more specific LM significantly improves performance for Croatian and Slovenian, even in the low data regime. We perform multiple analyses to shed light on the behavior of the models, and use visualization techniques to try and interpret the inner workings of our fine-tuned models.

The paper is organized as follows; first, in “Background and Related Work”, we start by providing a summary of offensive language detection, the use of different pre-trained language models, and intermediate training. In “Method and Datasets”, we describe our experimental pipeline, the dataset used, and model architecture. “Quantitative Results” presents our experiments and quantitatively answers our research questions. “Analysis and Qualitative Results” provides insight into the results using different analyses and some qualitative results. “Conclusion” concludes our contribution. The paper also contains an “Appendix” with additional detailed experimental results. The code and data splits for the experiments are made available on GitHub (https://github.com/EMBEDDIA/cross-lingual_training_for_offensive_language_detection).

## Background and related work

In this section we present an overview of the state of the art in offensive language detection, first reviewing defining the task and reviewing available datasets (Offensive Language Detection: Task and Datasets), and next describing current approaches to automatic detection, explaining their use of pre-trained language models (Automatic Detection and Pre-Trained Models). We then discuss approaches to multilinguality and cross-lingual training (Multilingual and Cross-lingual Approaches), and explain in detail the technique of intermediate training that we investigate here (Intermediate Training).

### Offensive language detection: task and datasets

Automatically detecting hate or offensive language is an increasingly popular task, with many public datasets, shared tasks, and models proposed to tackle it (see Schmidt & Wiegand, 2017; Poletto et al., 2020; Vidgen et al., 2020; Vidgen & Derczynski, 2020, for recent surveys). The exact definition of the categories annotated in these tasks varies, but they generally include threats, abuse, hate speech and offensive content. These terms are often used interchangeably, with some (particularly *hate speech*) often used to cover multiple categories. Exact definitions of the individual categories also vary with task and dataset. In this work, we use *offensive speech* as a generic term. The task is usually defined as a classification task, i.e., for a given text, determine if it is hate speech or not. Some tasks also try to classify at finer-grained levels and treat the task as a multi-class problem.

#### Datasets and languages

Most research on offensive language detection is monolingual, and English is still the most popular language, at least partly due to data availability (Wulczyn, Thain & Dixon, 2017; Golbeck et al., 2017; Davidson et al., 2017; Vidgen et al., 2020). Most data is collected from social media platforms (such as Twitter (Davidson et al., 2017), Facebook (Ljubešić, Fišer & Erjavec, 2019)), newspaper comments (Gao & Huang, 2017), YouTube (Obadimu et al., 2019), and Reddit (Qian et al., 2019). Lately, however, the focus has been shifting to other languages, with several shared tasks organized that cover other languages besides English, including EVALITA 2018 (Bai et al., 2018), GermEval 2018 (Wiegand, Siegel & Ruppenhofer, 2018) and SemEval 2019 Task 5 on Multilingual Detection of Hate Speech Against Immigrants and Women in Twitter (Basile et al., 2019). The OffensEval 2020 shared task (Zampieri et al., 2020a) also featured five languages: Arabic, Danish, English, Greek, Turkish. Some other non-English datasets for offensive language exist: Ibrohim & Budi (2018) annotated Indonesian tweets for abusive language, and Mubarak, Darwish & Magdy (2017) annotated abusive Arabic tweets. For Spanish, Plaza-Del-Arco et al. (2020) provide tweet collection annotated for misogyny and xenophobia, while Leite et al. (2020) provide toxic tweet collection in Brazilian Portuguese. Mathur et al. (2018) and Chopra et al. (2020) present data in Hinglish (spoken Hindi mixed with English written using the Roman script). The HASOC dataset (Mandl et al., 2019) is in English, German and Hindi, with both tweets and Facebook comments. Ljubešić, Erjavec & Fišer (2018) and Shekhar et al. (2020) provide data from Croatian newspaper comment sections.^1^ 1A comprehensive list of relevant datasets is available online at http://hatespeechdata.com/.

### Automatic detection and pre-trained models

A range of machine learning methods have been proposed to address the task, including logistic regression (Davidson et al., 2017; Pedersen, 2020), Naive Bayes (Shekhar et al., 2020), support vector machines (Salminen et al., 2018), and deep learning (DL) (Zampieri et al., 2020a). Most approach the problem as one of text classification, but some try to improve results via the addition of other data: Gao & Huang (2017) use the username and the title of the article as context to perform the task, while Farha & Magdy (2020) use a multi-task approach, and Salminen et al. (2020) develop a taxonomy of hate speech types with corresponding multiple models. Most recent approaches are DL-based, and a general trend in this direction is the use of pre-trained language models (LMs). The availability of large amounts of data, computational resources and the recently introduced Transformer architecture (Vaswani et al., 2017) have resulted in a large number of such pre-trained LMs, e.g., BERT (Devlin et al., 2019), RoBERTa (Liu et al., 2019) and others. These models are generally used by taking the pre-trained LM model weights as initialization, adding a task-specific classifier layer on top, and fine-tuning it using task-specific data. Variants of this approach have been shown to achieve the state of the art performance on multiple tasks like question-answering (Rajpurkar et al., 2016), the GLUE (Wang et al., 2018) and SuperGLUE (Wang et al., 2019b) benchmarks, as well as hate speech detection (see e.g., Liu, Li & Zou, 2019). In the OffensEval-2020 shared task (Zampieri et al., 2020a), most of the best-performing models use a variant of this approach.

### Multilingual and cross-lingual approaches

All these approaches, however, rely on suitable labeled training datasets in the target language. As explained in “Offensive Language Detection: Task and Datasets”, language coverage is increasing, but no datasets currently give (or can hope to give) resources for all languages, and any work in less-resourced languages will therefore require the development of new datasets from scratch. There is therefore significant interest in *cross-lingual* approaches to hate speech identification, in which a model for a chosen *target* language is trained using data in one or more different, better-resourced *source* languages.

Basile & Rubagotti (2018) conduct cross-lingual experiments between Italian and English on the EVALITA 2018 misogyny identification task, using the so-called *bleaching* approach (van der Goot et al., 2018), which aims to transform lexical strings into a set of abstract features in order to represent textual data in a language-agnostic way. While this approach shows a drop in performance in a monolingual setting, it outperforms the standard lexical approaches in a cross-lingual setting. More recent work uses neural networks: Pamungkas & Patti (2019) use a LSTM joint-learning model with multilingual MUSE embeddings, which are trained from parallel corpora in order to give cross-lingual representations (Lample et al., 2018). This showed improvement in a cross-lingual setting over a SVM with unigram features. However, cross-lingual models generally seem to perform worse than monolingual ones. Leite et al. (2020) tested monolingual and cross-lingual models based on multilingual BERT on Spanish and Portuguese data; the monolingual models outperformed their cross-lingual counterparts. Schneider et al. (2018) used multilingual MUSE embeddings to extend the GermEval 2018 German training set with more English data, but saw no improvement in performance. Stappen, Brunn & Schuller (2020) extended the original XLM architecture to a cross-lingual setting, and evaluated it in zero-shot (i.e., without any data in the target language) and few-shot (small amounts of target language data) settings, and found that even a small amount of target language data substantially improves model performance over the zero-shot setting.

Several questions remain unanswered, though. First, it is not yet clear how general this performance drop is across languages; Stappen, Brunn & Schuller (2020), for example, look at only one language pair, namely English and Spanish. In this paper, we therefore examine a broader range of languages. Another is the effect of the pre-trained LM used. Most current cross-lingual approaches are based on multilingual versions of the pre-trained LMs introduced above, such as multilingual BERT (mBERT, Devlin et al., 2019) and XLM-R (Conneau et al., 2020); as these are pre-trained on large multilingual corpora, their representations can transfer well between the languages seen in pre-training, and cross-lingual effects within these can be achieved by fine-tuning on a source language dataset and testing on a different target language. However, while these LMs perform reasonably well across a range of languages and tasks, they perform less well on a given domain or language than a model pre-trained for that specific domain (e.g., Lee et al., 2020, for biomedicine) or language (e.g., Martin et al., 2020, for French). Ulčar & Robnik-Šikonja (2020) provide two tri-lingual BERT models, FinEstBERT (Finnish/Estonian/English) and CroSloEngualBERT (Croatian/Slovenian/English), and show that they perform better in those languages than the more general mBERT on several tasks like NER, POS-tagging and dependency parsing. We might therefore expect LMs with more specific language combinations to perform better at cross-lingual transfer within those combinations, and this is another question we investigate here.

### Intermediate training

Another question is the effect of the choice and combination of source vs target language data when fine-tuning the pre-trained LM. The general mechanism in use here is often called *intermediate training*: starting with a pre-trained LM, first training on a similar source (or rather, in this setting, *intermediate*) task, and only then training on the desired target task. Most work in this direction examines the effect of intermediate training on a source task different from the target task (Yogatama et al., 2019; Wang et al., 2019a; Pruksachatkun et al., 2020; Vu et al., 2020). Yogatama et al. (2019) explore the transferability of linguistic knowledge in the LM to the target task: while some knowledge is transferred, fine-tuning is still needed to perform the target task, and the fine-tuned model is less transferable to the same task on different datasets. Wang et al. (2019a) conducted 17 instances of intermediate training on ELMo and BERT models on the GLUE benchmark tasks, finding that intermediate training doesn’t always help with target tasks. Surprisingly, they found no clear correlation between the intermediate task data size and fine-tuned target task performance. Pruksachatkun et al. (2020) also performed an extensive study of intermediate training using RoBERTa (Liu et al., 2019); consistent with Wang et al. (2019a), they also found no impact of intermediate task dataset size. In general, having high-level inference (e.g., co-reference resolution) and commonsense reasoning (e.g., QA) tasks as the intermediate task is helpful. In contrast to other work, Vu et al. (2020) show that intermediate training has a more significant effect on performance, and tested different settings to understand the impact of intermediate and target dataset size. The performance gain is highest when there is limited target training data; and the transferability of knowledge from intermediate to the target task is more dependent on the similarity between the intermediate and target tasks and datasets. Pelicon et al. (2020) used a sentiment classification task as intermediate task to boost the performance of the target task of news sentiment classification, with consistent findings. Lin et al. (2019) proposed a systematic way to transfer knowledge from one language to another, via a mechanism to select the best language pair for the transfer of knowledge.

In the domain of offensive language detection, Stappen, Brunn & Schuller (2020)’s cross-lingual experiments (see “Multilingual and Cross-lingual Approaches” above) can also be seen as an example of intermediate training, first fine-tuning with data in a language that was different from the target language, and then with differing amounts of data in the target language. They found that performance improves only in the case of small amounts of target data. As noted above, though, they investigated only one language pair (English/Spanish), and used only a general mBERT LM. Here, we attempt a more systematic and wider investigation of different intermediate training regimes, with different language pairs, and different pre-trained LMs.

## Method and datasets

In this study, we investigate the effectiveness of cross-lingual training for the problem of hate speech detection. This problem can be modeled as a classification task, formally stated as follows.

Let

}{}{\rm NN}:{{\bf X}_l} \to C

represent a classifier able to map from the space of text representations (e.g., byte pair encoded inputs) **X**_*l*_ in a given language *l* to the set of possible classes *C*. The purpose of this work is to explore the predictive performance of NN in a cross-lingual setting. Formally, we explore the performance of NN when trained on the space **X**_*a*_ and tested on **X**_*b*_, where *a* and *b* represent two different languages.

In this section, we describe our experimental setup, datasets, the details of our classification model architecture and optimization, and the evaluation metrics used.

### Experimental pipeline

**Figure 1:**
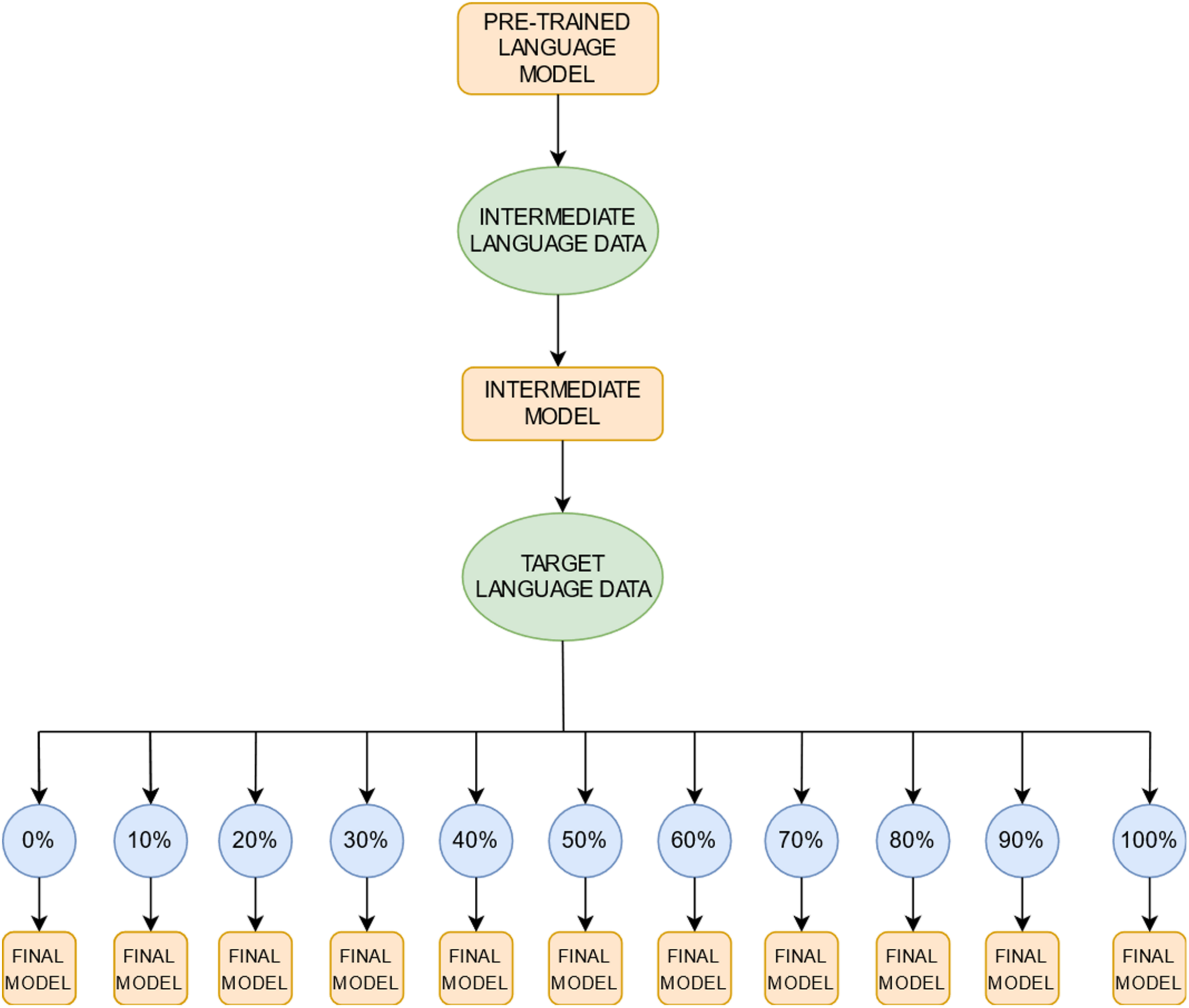
A schematic illustration of the training regime. We first select a *pre-trained* language model; further train it on data in one or more *intermediate* non-target languages to produce an *intermediate* model; then fine-tune the result by progressively adding data in the target language to produce the *final* model with which to evaluate performance. We progressively add data in the target language in 10% increments; the blue circles represent the proportion of target language data we use for training the final models. The step size of 10% was chosen arbitrarily. Note that the 0% setting presents the *zero-shot learning* setting where no target language data is used for fine-tuning and the intermediate model is evaluated directly on the target language data.

Our experimental pipeline (Fig. 1) consists of three steps: selection of a pretrained language model (LM), intermediate-task training on data in one or more non-target languages, and fine-tuning on a single target-language task. In the last fine-tuning step, we test the effect of variable amounts of target-language training data.

#### Language model

In order to investigate the effect of the pre-trained LM properties, we use two multi-lingual transformer based models: mBERT, a general model with 104 languages (Devlin et al., 2019), and CroSloEngual BERT, hereafter cseBERT, a much more specific model with only three languages (Ulčar & Robnik-Šikonja, 2020). All the languages used in the experiments are present in mBERT; three languages (Croatian, Slovenian and English) are present in cseBERT, allowing us to compare its effect on those and on others not included in its pre-training.

#### Intermediate training

In this step, we perform intermediate-task training of the model on a classification task in one or more non-target languages. We focus on three different languages for intermediate training, namely English, Slovenian and Arabic. English and Slovenian are used because they are used in both mBERT and cseBERT; use Latin script, common for all languages except Arabic; and give two points for comparison of language similarity (Slovenian is more similar to Croatian and less similar to German; English is more similar to German and less to Croatian, as discussed in “Introduction”). Finally, we include Arabic as it is the most dissimilar from all other languages used here, in terms of both linguistic and orthographic features, and is present in mBERT but not in cseBERT. We also test the use of intermediate training on all the languages except for the target language, and call this the *leave-one-(language-)out* (LOO) setting.

#### Target task fine-tuning

In the final step, we fine-tune our model on the target language task dataset following the standard procedure (Devlin et al., 2019). Depending on the configuration of the first two steps, the target task performance can then be observed with the different LMs, and with and without the different intermediate training variants.

#### Data hunger of the model

To observe how data availability influences the performance on the target language task, we gradually increase the amount of training data for the fine-tuning, from 0% target data (the zero-transfer setting) to 100% target data (the ideal fully-resourced scenario) in steps of 10%. We use this increasing data regime to investigate the following questions. First, does having a better pre-trained LM reduce the amount of target data needed to achieve good performance? Second, to what extent can intermediate training on another language compensate for unavailability of target language data (which would be especially valuable for less-resourced languages)? Last but not least, we test whether training in intermediate language(s) can boost the performance compared to training only in the target language.

### Datasets

We used hate speech and offensive language datasets in five different languages—English, Arabic, Croatian, Slovenian and German (see Table 1)—for intermediate training and fine-tuning:^2^ 2All the datasets used in this study were gathered in the course of other studies. For Slovenian the data is not public, but is available upon request from the original authors; for all other languages the datasets are publicly available (see cited references for details), and our GitHub repository (https://github.com/EMBEDDIA/cross-lingual_training_for_offensive_language_detection) provides exact data splits used in our study.

- **Croatian: 24sata** (Shekhar et al., 2020, Pollak et al., 2021). This dataset contains reader comments from the Croatian online news media platform 24sata (https://www.24sata.hr/). Each comment is labeled according to 8 rules covering Disallowed content (Spam), Threats, Hate speech, Obscenity, Deception & trolling, Vulgarity, Language, Abuse (see Shekhar et al., 2020, for annotation schema details). In this study we used only the Hate speech label, taking all comments without that label as non-hate speech.
- **English: OffensEval 2019** (Zampieri et al., 2019a). This dataset contains Twitter posts that are labeled according to a three-level annotation scheme. On the first level, each tweet is labeled as either offensive or not offensive. Those labeled as offensive are then annotated on a second level as either targeted (i.e., directed at a particular individual or group) or untargeted (i.e., containing general profanity). Those labeled as targeted are further labeled on a third level as directed towards a specific individual, group or other entity. For our task we use only the first level (offensive/non-offensive).
- **Slovenian: FRENK** (Ljubešić, Fišer & Erjavec, 2019). This dataset contains Facebook posts, and uses a 3-label annotation schema, where each post is annotated as Acceptable, Other offensive (i.e., containing general profanity), Background offensive (i.e., containing insults or profanity targeted at a specific group). The dataset is divided in two parts, one on the topic of migrants and migrations and the other on the topic of LGBT communities. Both parts were collected by the same group following the same procedure. We used both migrant and LGBT datasets together and combine all offensive classes into one class.
- **German: GermEval 2018** (Wiegand, Siegel & Ruppenhofer, 2018). This dataset contains Twitter posts labeled on two levels. On the first level, each tweet is labeled as either Offensive or Other. Those labeled as Offensive are then labeled on the second level as either Profanity, Abuse or Insult. For our classification task, we use only the first level (offensive/non-offensive).
- **Arabic: OffensEval 2020** (Zampieri et al., 2020a). This dataset contains Twitter posts, gathered and annotated by the same team as the OffensEval 2019 English Dataset (see above); it uses the same annotation schema and we treat it in the same way.

**Table 1:** Original dataset sizes and label distribution.

Language	Source	Original size	Not-offensive proportion (%)	Offensive proportion (%)
Croatian (Shekhar et al., 2020)	News comment	99,246	50	50
Slovenian (Ljubešić, Fišer & Erjavec, 2019)	Facebook	12,400	46	54
English (Zampieri et al., 2019a)	Twitter	13,240	67	33
German (Wiegand, Siegel & Ruppenhofer, 2018)	Twitter	8,884	67	33
Arabic (Zampieri et al., 2020a)	Twitter	7,839	80	20

Although all the datasets were annotated for hate speech or offensive language detection tasks, the authors employed different annotation schemes due to their domain and specific purposes and phenomena. This reflects the current situation, in which a large number of labeled hate speech datasets are freely available for different languages, but do not share a common annotation procedure. These discrepancies, albeit small, can potentially impact a model’s ability to properly converge if one were trying to boost performance using data across several datasets and languages. In this way, our experimental setting reflects this real-world scenario and provides a realistic estimation of the models’ behavior.

To deal with the differences in annotations, we consolidated the annotation schemas of different datasets so as to model the problem as a similar binary classification task in each case. For this purpose, we use the first-level annotations of the English, German and Arabic datasets, which label the documents as either offensive or not offensive. For the Slovenian dataset, in which offensive posts are labeled in several categories on one level, we combine the different offensive categories into one offensive class. For the Croatian dataset only the hate speech label is used, as the other categories represent different reasons for blocking comments which may not necessarily include offensive language of any kind.

To minimize the effect of dataset size on the performance of the model, we use the same amount of training data for each language. We reduced the size of all datasets to the size of the smallest dataset in the set, namely the Arabic dataset with 7839 instances, while keeping the class balance the same. We split the resulting datasets into training, validation and test sets in the proportion 80-10-10.^3^ 3The splits for the English, German, Croatian and Arabic datasets are available on the GitHub repository (https://github.com/EMBEDDIA/cross-lingual_training_for_offensive_language_detection). The code for Slovenian data splits is provided on the same GitHub, however the data itself should be obtained from Ljubešić, Fišer & Erjavec (2019).

### Models and optimization

We perform the whole three-step experiment described in “Experimental Pipeline” using a BERT-based language model (mBERT or cseBERT). The representation of the (CLS) token from the last layer of the BERT language model is used as a sentence representation, and passed to a further linear layer with a softmax activation function to perform the classification. The whole model is jointly trained on the downstream task of hate speech detection. Fine-tuning is performed end-to-end. All models were trained for maximum 4 epochs with batch size 16. The best model is selected based on the validation score. We used the Adam optimizer with the learning rate of 2 × 10^−5^ and learning rate warmup over the first 10% of the training instances. For regularization purposes we used weight decay rate set to 0.01. The same optimization process was used for both the intermediate training and the fine-tuning steps of our training setup. We perform the training of the models using the HuggingFace Transformers library (Wolf et al., 2020). To perform matrix operations in an efficient manner we ensured all inputs were of the same length, first tokenizing all inputs and then setting their maximum length to 256 tokens. Sequences larger than this maximum were shortened, while longer sequences were zero-padded. As is standard with the BERT architecture, each of these models was pre-trained with minimal text preprocessing and comes with its own tokenizer which tokenizes text at word and sub-word levels. We applied the same procedure in the intermediate learning and fine-tuning phases, tokenizing the text input using the default tokenizers that were trained with the mBERT and cseBERT models, with no additional text pre-processing.

### Evaluation metrics

Due to imbalance in the dataset, we follow the standard evaluation metrics used in OffensEval (Zampieri et al., 2019a) and report the macro-averaged F1 score. To counteract the effect of random initialization of the model, we trained models with three different random seeds and report mean and standard deviations of F1 scores. To qualify the performance with increasing data, we report the area-under-curve (AUC) with respect to the F1-score and data size. For more detailed evaluation information, we also provide two other standard evaluation metrics, macro-averaged recall and precision, again reported as mean and standard deviation over the three training runs with different random seeds. For readability purposes, we present these results in the “Appendix”.

To test for statistical significance of differences between results, we use the Mann–Whitney *U* test with a significance level of 0.05. We choose this non-parametric test as it makes no assumptions about normality of distribution and is suitable to be used with a small number of samples (3 runs of each experiment in our case).

## Quantitative results

In this section, we present quantitative results, and in particular answer the research questions presented in “Introduction” concerning the effects of pre-trained model selection, intermediate training (using one or more additional languages), and amount of target language training data.

### Monolingual results

To provide points of comparison, we first give results for the standard monolingual case in which all target-language data is assumed to be available and used in fine-tuning, with no intermediate training; together with baseline results based on the majority class and on random model weight initialization. For the majority class baseline, we simply give all test set examples the same label as the majority class in the training set data. For the random initialization baseline, we attach the pre-trained LM to the randomly initialized classifier layer.

Table 2 shows these results for both mBERT and cseBERT. Random initialization of the model is in most cases similar to the majority class baseline and has very high standard deviation; it allows us to explicitly examine the effect of fine-tuning. As expected, after fine-tuning the model on the entire target-language dataset, the performance of the model is always substantially higher than the majority class and random initialization baselines (for both mBERT and cseBERT). The highest gain over the majority class baseline is observed for Arabic with mBERT, and for Slovenian with cseBERT. The best performances for each language (see bold columns in Table 2) are overall of a similar level to those reported in other work, giving us confidence that we are experimenting with models which approach the monolingual state of the art. Please note, however, that due to resizing of the datasets (as explained in “Datasets”) our results were obtained on different train-validation-test splits than the results from related work and are therefore not directly comparable.

**Table 2:** Comparison of mBERT and cseBERT, fine-tuning on all training data in the target language only (no intermediate training), together with the majority class and randomly initialized models baselines. Values are shown as macro-averaged F1-score with standard deviation. Bold indicates the best performance for each language; † indicates that the difference is statistically significant based on the Mann–Whitney *U* test. For comparison also the following state-of-the-art (SOTA) results are provided: Shekhar et al. (2020)^1^, Miok et al. (2021)^2^, Zampieri et al. (2019b)^3^, Struß et al. (2019)^4^, Zampieri et al. (2020b)^5^. Note however that the SOTA results are based on different data splits. For macro-averaged precision and recall scores, see Tables 10 and 11.

Language	Majority class	mBERT		cseBERT		SOTA
		Random init.	Fine-tuned	Random init.	Fine-tuned	
Croatian	43.72	49.99_3.30_	71.10_1.42_	45.85_4.83_	†**74.98**_1.06_	71.78^1^
Slovenian	34.83	44.33_6.44_	72.73_0.36_	44.94_3.27_	†**76.11**_0.58_	68.60^2^
English	41.89	47.72_3.57_	76.63_1.15_	42.32_9.09_	**77.10**_1.34_	82.90^3^
German	39.46	31.19_4.89_	†**75.90**_0.38_	40.96_10.60_	73.98_0.98_	76.95^4^
Arabic	44.32	50.13_1.91_	†**84.62**_0.19_	45.73_9.26_	76.01_0.61_	90.17^5^

### Effect of pre-trained LM

Comparing the performance of mBERT and cseBERT (Fine-tuned columns in Table 2), we observe that using cseBERT always outperforms mBERT for the languages cseBERT is pre-trained on (ΔF1 +3.88 Croatian, +3.38 Slovenian, +0.47 English); but performance decreases for languages not used in cseBERT pre-training (ΔF1 −1.92 German, −8.61 Arabic). For English, mBERT and cseBERT performances are very similar. The improvement in performance in Slovenian and Croatian using cseBERT, which was pre-trained with higher quality resources for Slovenian and Croatian, is consistent with the findings of the authors of cseBERT (Ulčar & Robnik-Šikonja, 2020) on a range of tasks. This also suggests that improving the pre-trained models especially benefits less-resourced languages like Slovenian and Croatian. The decrease in performance for Arabic is higher than that for German. This could be attributed to the fact that cseBERT is pre-trained only on languages in Latin script, perhaps resulting in little overlap in sub-word token vocabulary with Arabic. For German, some sub-words will be shared between the languages in the pre-training and testing phases (see “Analysis of Vocabulary Coverage”). However, as the performance of cseBERT is still decent on languages not used in pre-training, the fine-tuning step seems of high importance and the pre-training phase plays only a limited role in these cases.

### Effect of intermediate training

As a next research question, we asked whether intermediate training on different languages can boost the classifier performance on the target language. First, we evaluate the effect of intermediate training without fine-tuning on the target language training data: the *zero-shot transfer* scenario. As Table 3 shows, for most cases, intermediate training gives substantial increases over the baseline, except for German and Arabic with cseBERT. This shows that the model learns some useful knowledge from intermediate training and transfers it to the target language task: performances are reasonable in many cases, although they do not reach the levels of the monolingual results of Table 2, confirming the findings of Stappen, Brunn & Schuller (2020) and Leite et al. (2020). Again, we see that cseBERT gives better results for its languages (e.g., transfer from English to Croatian and Slovenian) than mBERT, while mBERT does better when Arabic is the target. Encouraged by this result, we test the effect of intermediate training in the well-resourced scenario: fine-tuning the intermediate trained model using all target language task data. Table 4 shows the results of fine-tuning only on target language data (repeated from Table 2), compared to the use of intermediate training using English, Slovenian and Arabic respectively, before fine-tuning in the target language as before. In the last column (LOO+TGT), we include all languages except the target language (LOO) in the intermediate training step.

In most cases, adding one or more languages improves the results (the exceptions being the English target language for mBERT and German target language for cseBERT). However, the gain in performance is not large. In the case of mBERT, the largest gain is achieved for Slovenian by using LOO intermediate training (ΔF1 +2.26); followed by Arabic with Slovenian intermediate training (ΔF1 +1.13), Croatian with Slovenian intermediate training (ΔF1 +1.02), and German with English intermediate training (ΔF1 +0.17). English performance decreases with all the intermediate training variants. Using cseBERT shows a similar trend, where the largest gain is for Arabic (ΔF1 +2.52), then Croatian (ΔF1 +1.56), Slovenian (ΔF1 +0.67) and English (ΔF1 +0.63), while performance for German decreases (ΔF1 −2.38). However, the gains using LOO (all available non-target language data) are always either the highest or very close to it, suggesting that this is the most useful practical approach in most cases. There is no conclusive evidence of the role played by the script; for example, Arabic intermediate training improves the performance of Croatian and Slovenian with mBERT while the performance decreases for English and German. Overall it seems that although intermediate training can provide gains, they are relatively small in most cases: whenever there is a large amount of data available for a task, training on the target task is likely to be sufficient to achieve optimal performance on that dataset, and using intermediate training in a different language(s) is unlikely to give significant gains.

**Table 3:** Comparison of intermediate training in a range of non-target languages in zero-shot transfer on the target language data, for mBERT (top) and cseBERT (bottom). TGT: random initialization (no intermediate training, no target fine-tuning). ENG/SLO/AR → TGT: Intermediate training on English/Slovenian/Arabic, then zero-shot transfer on the target language. LOO → TGT: Intermediate training on all non-target languages, then zero-shot transfer on the target language. Values are shown as macro-averaged F1-score with standard deviation. Bold indicates the best performance for each target language and arrows indicate increase/decrease compared to the randomly initialized baseline. For macro-averaged precision and recall scores, see Tables 12 and 13.

Target	TGT	ENG → TGT	SLO → TGT	AR → TGT	LOO → TGT
	**mBERT**				
Croatian	49.99_1.54_	↑60.30_1.02_	↑59.97_0.22_	↓47.98_0.46_	↑**62.83**_0.58_
Slovenian	44.33_1.45_	↑**59.57**_0.77_	*–*	↓35.55_0.88_	↑47.00_0.93_
English	47.72_0.90_	–	↓43.28_1.40_	↓44.11_0.21_	↑**49.07**_0.52_
German	**31.19**_1.82_	↓28.43_1.95_	*↓28.01*_*4.41*_	↓27.43_6.63_	↓27.72_9.72_
Arabic	50.13_2.90_	↓46.00_2.53_	↑**59.68**_2.43_	–	↑56.71_1.31_
	**cseBERT**				
Croatian	45.85_9.87_	↑**67.70**_0.34_	↑67.56_0.69_	↓44.51_0.97_	↑67.12_0.91_
Slovenian	44.94_1.47_	↑**63.98**_0.12_	–	↓34.34_0.28_	↑58.75_0.40_
English	42.32_14.15_	–	↑53.61_0.34_	↑44.67_1.42_	↑**60.42**_0.88_
German	**40.96**_5.52_	↓25.69_1.56_	↓26.20_0.00_	↓25.83_0.77_	↓26.63_0.00_
Arabic	**45.73**_6.40_	↓44.97_3.30_	↓44.97_4.54_	–	↓44.97_3.15_

**Table 4:** Comparison of intermediate training in a range of non-target languages, followed by fine-tuning on all target language data, for mBERT (top) and cseBERT (bottom). TGT: Only fine-tuned on target language (no intermediate training). ENG/SLO/AR → TGT: Intermediate training on English/Slovenian/Arabic, then fine-tuning on target language. LOO → TGT: Intermediate training on all non-target languages, then fine-tuning on target language. Values are shown as macro-averaged F1-score with standard deviation. Bold indicates the best performance for each target language and arrows indicate increase/decrease compared to the randomly initialized baseline. For macro-averaged precision and recall scores, see Tables 14 and 15.

Target	TGT	ENG → TGT	SLO → TGT	AR → TGT	LOO → TGT
	**mBERT**				
Croatian	71.10_1.42_	↑71.96_1.55_	↑**72.12**_0.48_	↑71.88_0.80_	↑71.43_0.30_
Slovenian	72.73_0.36_	↓72.33_1.07_	–	↑73.89_0.68_	↑**74.99**_1.07_
English	**76.63**_1.15_	–	↓74.05_1.01_	↓74.73_0.31_	↓76.09_1.04_
German	75.90_0.38_	↑**76.07**_0.15_	↓74.46_0.04_	↓74.90_1.16_	↓75.02_0.52_
Arabic	84.62_0.19_	↓84.07_0.45_	↑**85.75**_1.03_	–	↑85.56_0.53_
	**cseBERT**				
Croatian	74.98_1.06_	↑**76.54**_0.98_	↓74.93_0.42_	↑75.37_0.70_	↑76.00_0.59_
Slovenian	76.11_0.58_	↑**76.78**_0.34_	–	↓76.03_0.44_	↑76.42_0.31_
English	77.10_1.34_	–	↑77.12_0.82_	↓77.06_1.00_	↑**77.73**_0.35_
German	**73.98**_0.98_	↓71.60_1.09_	↓69.30_0.40_	↓70.50_0.20_	↓69.34_0.87_
Arabic	76.01_0.61_	↑76.43_0.36_	↑76.58_1.42_	–	↑**78.53**_1.26_

### Data hunger of the model

**Figure 2:**
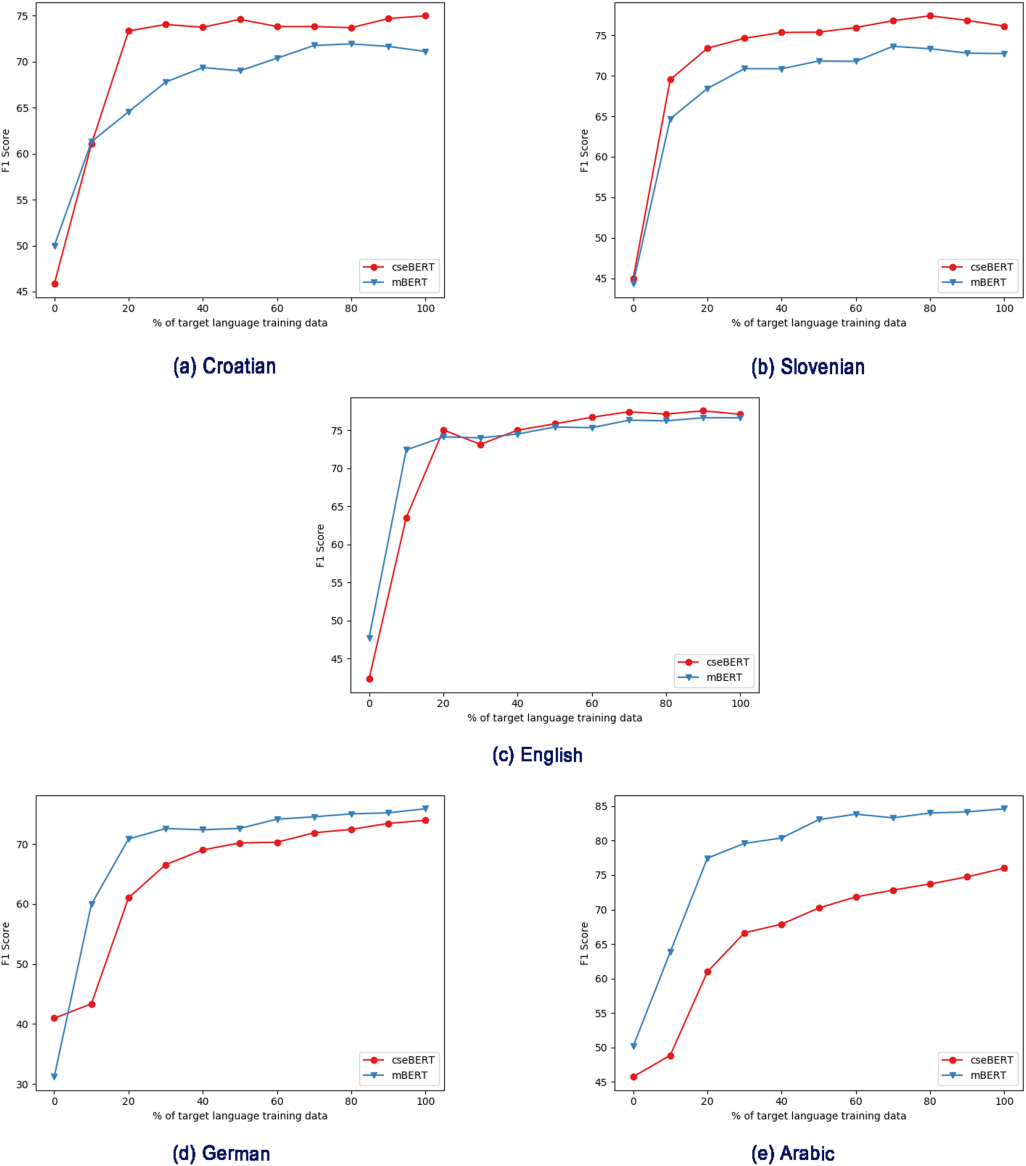
Effect of different pre-trained LMs (mBERT vs cseBERT), with varying amount of target language training data in the fine-tuning step, and no intermediate training. (A) Croatian, (B) Slovenian, (C) English, (D) German, (E) Arabic.

**Figure 3:**
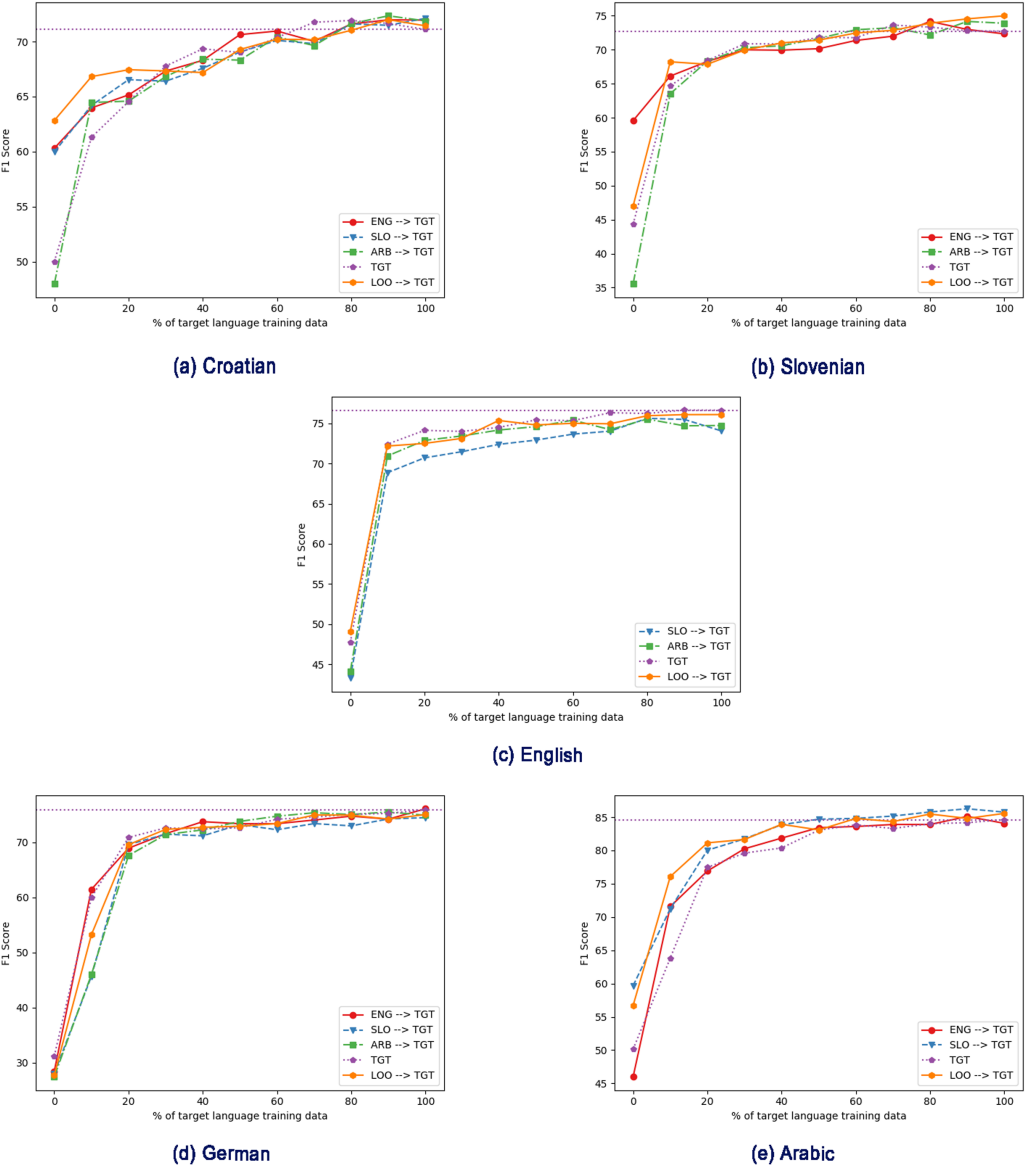
Effect of different intermediate training languages, with varying amount of target language training data in the fine-tuning step, using mBERT. TGT: Only fine-tuned on target language (no intermediate training). (A) Croatian, (B) Slovenian, (C) English, (D) German, (E) Arabic. ENG/SLO/AR → TGT: Intermediate training on English/Slovenian/Arabic, then fine-tuning on target language. LOO → TGT: Intermediate training on all non-target languages, then fine-tuning on target language.

**Figure 4:**
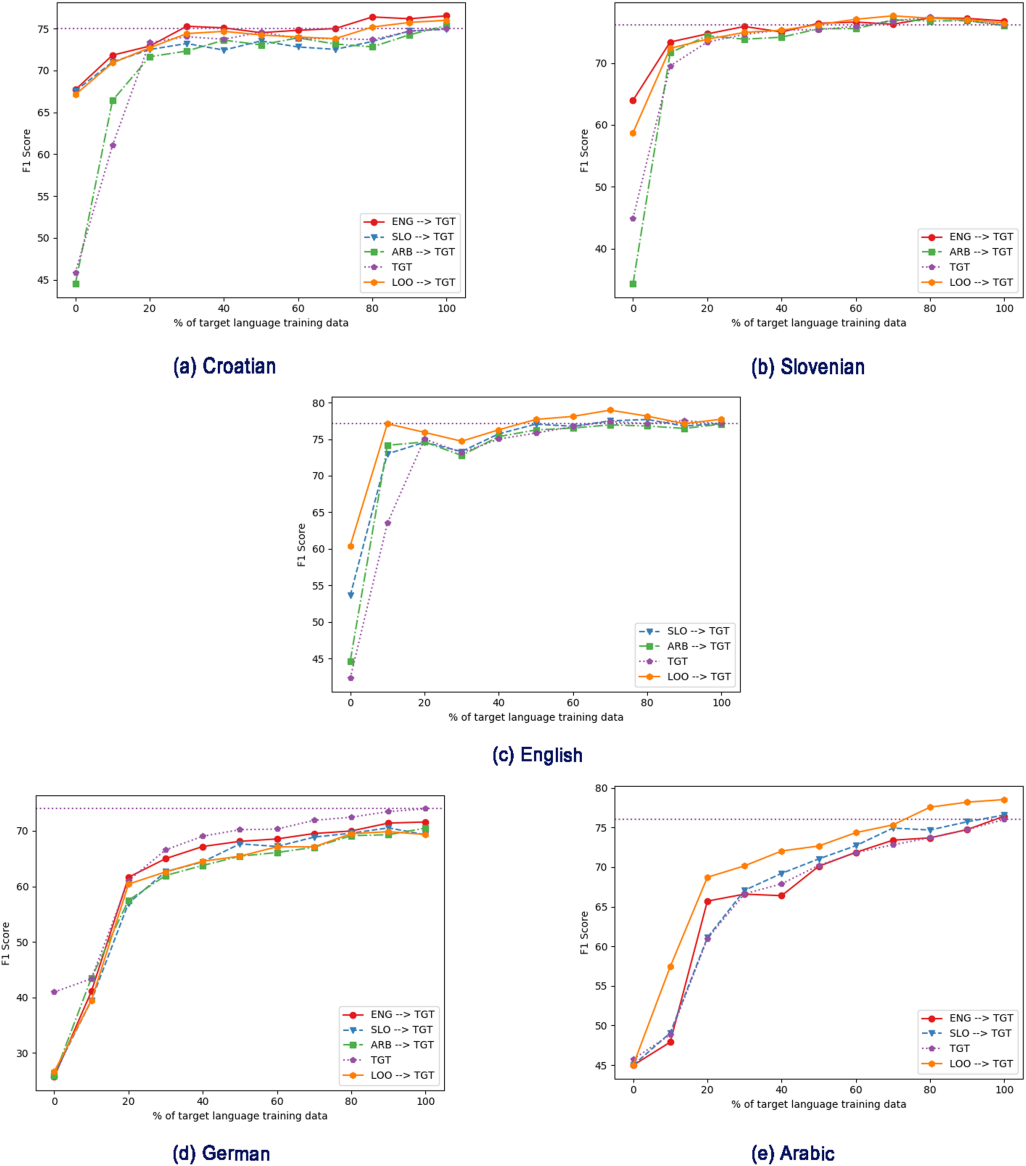
Effect of different intermediate training language with varying amount of target training data, using cseBERT. TGT: Only fine-tuned on target language (no intermediate training). (A) Croatian, (B) Slovenian, (C) English, (D) German, (E) Arabic. ENG/SLO/AR → TGT: Intermediate training on English/Slovenian/Arabic, then fine-tuning on target language. LOO → TGT: Intermediate training on all non-target languages, then fine-tuning on target language.

We next explore the effect of different amounts of training data, first in the monolingual, target-language-only case (Fig. 2), and then with intermediate training (Figs. 3 and 4).

Figure 2 shows the increasing data training regime without intermediate training, and shows a substantial difference between the performance with the mBERT and cseBERT LMs. With Croatian and Slovenian (the less-resourced languages on which cseBERT is trained), not only does cseBERT outperform mBERT (following the full-dataset results in Table 2), but performance is relatively high, and increase over mBERT is substantial, even with a very small amount of training data (e.g., 10%). On the other hand, for German and Arabic, mBERT outperforms cseBERT. For English, performance is similar, reconfirming the pattern from Table 2 that on English there is no large gain by using the cseBERT model.

Next, we apply the same regime of gradually increasing the amount of target-language fine-tuning data, but this time after using intermediate training (thus testing the scenario where we have large amounts of data in similar tasks in other languages but little in the target language). Figures 3 and 4 show the results for mBERT and cseBERT respectively, including results without intermediate training, for comparison. In most cases, for comparatively low amounts of target-language data (~10%), intermediate training improves the results compared to fine-tuning purely on the target task if it is done using all the non-target languages available (see Table 5). In this case, we observe statistically significant improvements in 6 out of 10 experimental settings: for Slovenian and Croatian (with both LM), English (with cseBERT) and Arabic (with mBERT). For the other 4 settings, the results slightly degrade but the differences are not statistically significant. For settings, when we used only one language for intermediate training, the results seem to be inconclusive.

**Table 5:** Comparison of mBERT and cseBERT with intermediate training using all non-target languages (LOO setting) and fine-tuning on only 10% training data in the target language. Values are shown as macro-averaged F1-scores. Differences marked with † are statistically significant. Bold indicates the best performance for each language.

Language	mBERT		cseBERT	
	TGT	LOO → TGT@10%	TGT	LOO → TGT@10%
Croatian	61.30	†**66.82**	61.04	†**70.91**
Slovenian	64.68	†**68.22**	69.52	†72.63
English	**72.40**	72.17	63.51	†**77.11**
German	**59.97**	53.20	**43.36**	39.64
Arabic	63.82	†**76.07**	48.84	**57.42**

However, when more target language data is available, the gains from intermediate training drop. In other words, intermediate training only helps when target-language data is scarce. We can also see that intermediate training does not always lead to improved performance (shown also in experiments in Table 4). For example, for Croatian, using intermediate training on mBERT with a large amount of data decreases performance, while with cseBERT the performance is consistently improved. For mBERT on English, using Slovenian data for intermediate training clearly decreases performance. For Slovenian and Arabic, performance improves in all intermediate training settings, even with the full amount of training data. For cseBERT and Arabic, we can see that the LOO setting brings important gains in the performance, which can be explained by the fact that the LOO setting contains training data in languages used in the cseBERT pre-training. For English and cseBERT, we can clearly see that the LOO intermediate training is very useful if we have less than 80% of target data available.

To quantify the overall gains, in Table 6 we report the area under the F1-score curve (AUC) as the target language dataset size varies from 0% to 100% (see Figs. 3 and 4). Overall, we see that intermediate training helps; the exceptions are German for both mBERT and cseBERT, and English when using mBERT. The highest gain can be observed for Arabic and Croatian with cseBERT (improving by ~4% and ~3%, respectively); both languages show gains with mBERT too, although smaller. The gain in Arabic strongly suggests that intermediate training helps even if scripts are different. For Slovenian when using cseBERT we also gain more than ~1% with intermediate training on English, and when using mBERT less than ~1% with LOO setting. For German, performance is inconsistent: with English intermediate training, performance drops by ~1%, and with Slovenian it improves by ~1%.

**Table 6:** Area Under the Curve (AUC) of F1-score as we vary amount of target language training data in the fine-tuning step from 0% to 100%, for different intermediate training languages. TGT: Only fine-tuned on target language (no intermediate training). ENG/SLO/AR → TGT: Intermediate training on English/Slovenian/Arabic, then fine-tuning on target language. LOO → TGT: Intermediate training on all non-target languages, then fine-tuning on target language. Bold indicates the best performance for each target language. Pairwise statistical tests for each training setting show statistically significant differences between mBERT and cseBERT results for all settings.

Target	TGT	ENG → TGT	SLO → TGT	AR → TGT	LOO → TGT
	**mBERT**				
Croatian	67.82_1.22_	↑68.61_0.78_	↑68.28_0.45_	↓67.66_0.24_	↑**68.86**_0.25_
Slovenian	69.67_0.73_	↑70.09_0.10_	–	↓69.16_0.24_	↑**70.32**_0.14_
English	**73.71**_0.32_	–	↓71.38_0.38_	↓72.52_0.01_	↓73.25_0.21_
German	**70.10**_0.50_	↓69.76_0.24_	↓67.51_0.55_	↓68.27_0.47_	↓68.95_1.37_
Arabic	78.70_0.16_	↑79.55_0.26_	↑81.63_0.47_	–	↑**81.64**_0.09_
	**cseBERT**				
Croatian	71.31_1.36_	↑**74.42**_0.19_	↑72.75_0.22_	↓71.12_0.39_	↑73.73_0.26_
Slovenian	73.57_0.29_	↑**75.31**_0.17_	–	↓73.08_0.33_	↑74.91_0.13_
English	73.10_0.80_	–	↑74.78_0.13_	↑74.08_0.51_	↑**76.32**_0.24_
German	**65.59**_0.71_	↓63.11_0.46_	↓61.51_0.43_	↓61.19_0.81_	↓61.40_0.16_
Arabic	66.85_0.94_	↑67.11_0.37_	↑67.63_0.77_	–	↑**70.82**_0.85_

In terms of cseBERT and mBERT comparison, the results are consistent with those in Table 2: cseBERT improves over mBERT for the languages it is trained on (Croatian and Slovenian). For Arabic there is a large performance gap (~11%) between mBERT and cseBERT. We hypothesize that this is due to vocabulary: the cseBERT model sees no Arabic words in pre-training. cseBERT also doesn’t know German words, but the performance drop for German is much lower than for Arabic (less than ~5%); therefore we hypothesize that due to the Latin script of German and relative closeness to English and Slovenian, the sub-word tokenization provides some common vocabulary. German is closer to English as both are Germanic languages, but German also had a historically big influence on the evolution of the Slovenian language, therefore, there are bound to be words with similar roots.

With this quantitative analysis, we have shown that cross-lingual transfer can be effective for the offensive speech detection task, giving results with good performance even with small amounts of target language data. Using a better language-specific multilingual BERT (here, cseBERT) improves performance for languages that are less well represented in the standard mBERT model, and requires comparatively less target language data to achieve close to optimal performance. However, using different language task data as intermediate training doesn’t improve the performance in all cases; but when the target-language dataset size is small, intermediate training does give improvements.

## Analysis and qualitative results

In this section, we take a closer look at the performance of the models. In “Analysis of Misclassification”, we examine how mBERT and cseBERT differ in their mistakes, with a per-example analysis of several trained models to explore how the misclassifications change with different pre-trained language models. In “Analysis of Classifier Confidence”, we go further and examine misclassifications and different kinds of example via patterns in the confidence of the model outputs. While in “Analysis of Vocabulary Coverage”, we look at the vocabulary coverage and compare it with the model’s performance.

### Analysis of misclassification

We analyze the performance of mBERT and cseBERT using misclassified examples, aiming to explore how the space of misclassified samples behaves and changes when we change the underlying language model. Although standard performance metrics give us some idea of the models’ performance varies on different classes, they do not provide any insight into the performance across particular examples. For example, two models may achieve the same overall accuracy score yet may misclassify completely different examples.

**Figure 5:**
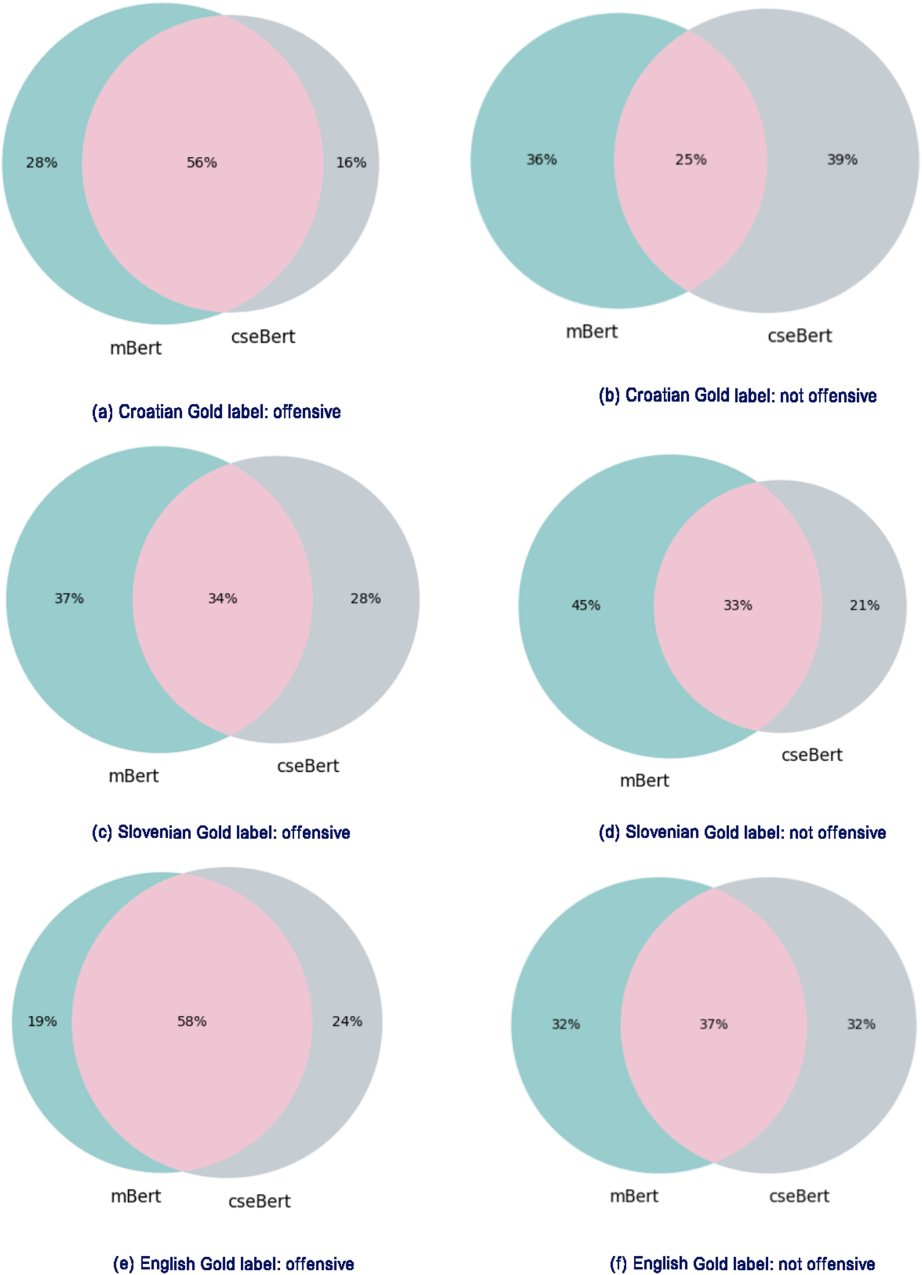
Comparison of misclassified examples for the mBERT and cseBERT models trained on 100%data with no intermediate learning step. (A) Croatian Gold label: offensive; (B) Croatian Gold label: not offensive; (C) Slovenian Gold label: offensive; (D) Slovenian Gold label: not offensive; (E) English Gold label: offensive; (F) English Gold label: not offensive. Figures on the left show misclassified examples with the ‘offensive’ gold label; on the right, misclassified examples with the ‘not offensive’ gold label. Green subsets: misclassified by mBERT but correctly classified by cseBERT. Grey subsets: misclassified by cseBERT but correctly classified by mBERT. Violet subsets: misclassified by both models.

The analysis is performed on the three languages of cseBERT (Croatian, Slovenian and English); for each language, we perform a pair-wise comparison of mBERT and cseBERT model outputs. All compared models were trained using 100% of target language training data without any intermediate training (corresponding to the quantitative results in Table 2). Figure 5 presents, for each comparison, the percentage of misclassified test set examples in the form of Venn diagrams, one for ‘offensive’ examples and one for ‘not offensive’ (according to the gold-standard labels). The different subsets in the diagrams show the proportions misclassified by mBERT alone, by cseBERT alone, and by both models together.

Figures 5E and 5F show that mBERT and cseBERT perform similarly for English. The subset of examples misclassified by both models is relatively large, covering 58% of the offensive and 37% of not-offensive examples. The other two subsets are of similar size: each model corrected some mistakes from the other model but made a similar number of mistakes on other examples. The results seem to be more in favor of cseBERT for the Slovenian and Croatian languages (see Figs. 5A–5D). Fewer examples are misclassified by cseBERT than mBERT, except for the Croatian ‘not offensive’ case. For these two languages, the proportion of shared misclassified examples is also much lower than for English, in all settings except for the Croatian ‘offensive’ examples (56%), where it is close to (but still lower than) the ‘offensive’ English examples.

These results show that while cseBERT does not seem to have any advantage for English, it performs substantially better for Slovenian and Croatian, in line with the quantitative results of Table 2. For these languages, it correctly classifies a range of examples for which mBERT makes incorrect predictions. Furthermore, the reduced number of the Slovenian and Croatian shared misclassifications may suggest that these models have gained different knowledge during their pre-training phases. These results show great promise for using these two models in tandem, e.g., as part of an ensemble, to produce higher quality models for hate speech detection in Slovenian and Croatian.

### Analysis of classifier confidence

**Figure 6:**
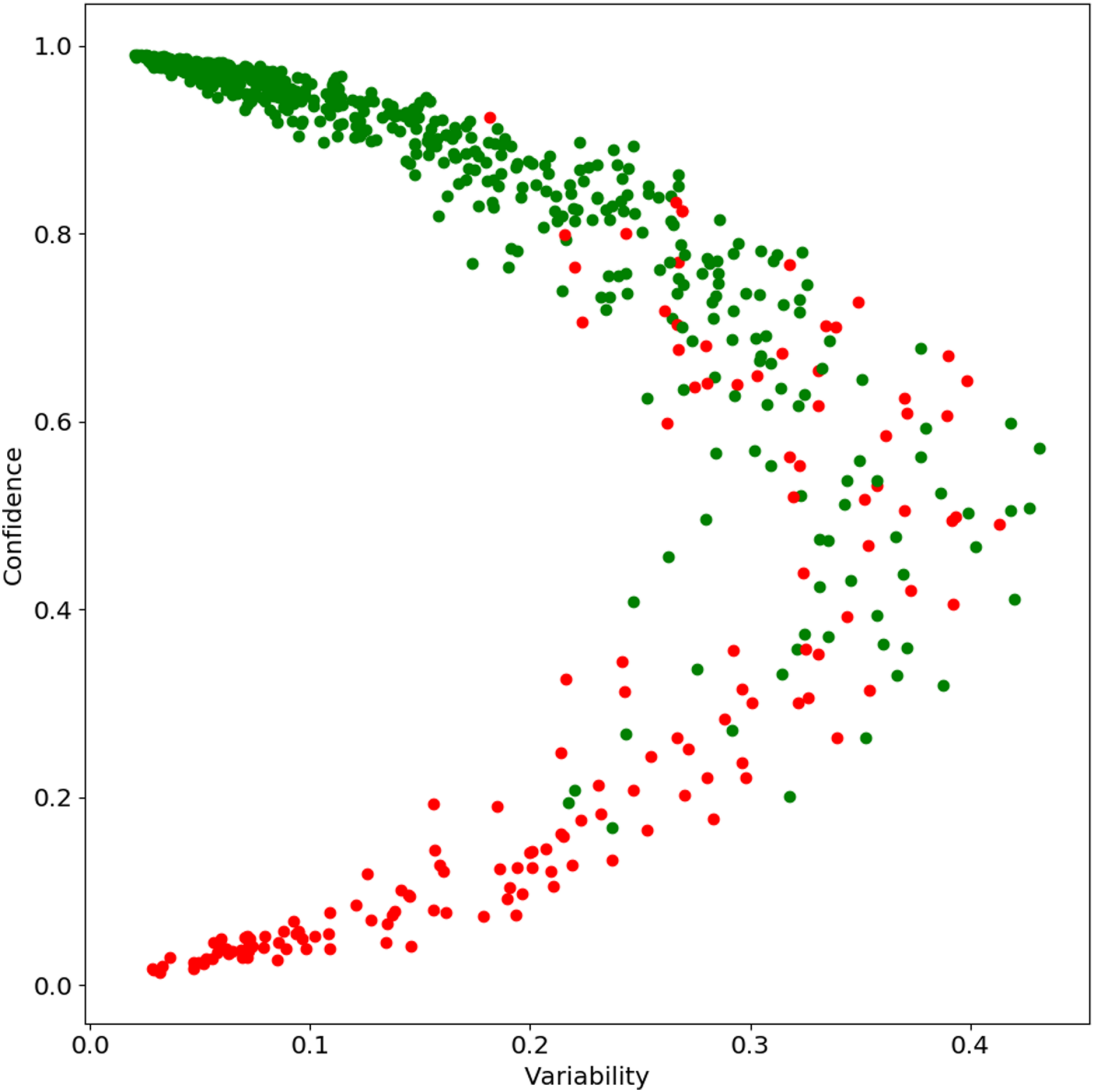
Confidence Score for English data: green when example is correct and red when example is incorrect by the best selected model with 100% data.

In this section, we look for patterns in the outputs based on the classifier’s confidence. Specifically, we analyze how “true” label confidence varies as the model is trained using more and more data (see data hunger analysis in “Quantitative Results”). Formally, for a test instance (*x*_*i*_) on the *j*% of the target data at the *k*th epoch, we looked at the correct label probability for all trained models. The *confidence* of the classifier is defined as the mean of the correct label probabilities and the *variability* the standard deviation. We analyzed the *confidence* and *variability* together to find the overall behavior of the test data Following Swayamdipta et al. (2020), we plot *confidence* and *variability* on the *Y*-axis and *X*-axis respectively. Please note that Swayamdipta et al. (2020) calculated confidence and variability over epochs; we used both changes over the data size and epochs. Figure 6 shows the confidence-variability plot for the English data; we found a similar pattern for other languages. As we can see from Fig. 6, there are three groups of instances. First, those for which the classifier is correct and has very high confidence and low variability, i.e., “easy” examples. Second, those where classifier confidence is close to 0.5 and has high variability, i.e., “ambiguous” examples. And third, where the classifier has very low confidence and variability for the true label, i.e., “hard” examples.

To further analyze these three categories, we manually inspected some examples and tried to understand what makes them easy, ambiguous, or hard for the classifier to classify. We present some of these examples in Tables 7–9. Most easy examples are characterized by specific offensive words or phrases. For example, in Table 7, the first example has “Nigga ware da”, and the second example has only socially accepted words. In the hard category, many examples are cases where it is hard to identify from the sentence alone whether it is offensive or not, without some form of context. The classifier generally made mistakes in classifying such instances. For example, in Table 7, one example needs context in the form of the URL, and the other one is dependent on the comment it is replying to. The ambiguous category is perhaps the most interesting: in many cases, the annotation appears to be wrong, and in others another label is equally possible. For such examples, we have provided the potentially correct labels in the tables. The classifier seems to work inconsistently for these instances; we believe this is because these instances have patterns similar to the class opposite to their gold label. Please note that these three classes are not rigidly defined: several examples could belong to other classes. In particular, there are overlaps between the hard and ambiguous classes: in many cases the gold labels appear to be wrong for “hard” examples, and “ambiguous” examples require context. However, most such overlaps occur at the boundaries of the classes.

**Table 7:** English examples from the test set belonging to different regions in the confidence-variability plot with gold labels and classifier labels. Examples which seem to have been assigned an incorrect gold label are given a possible correct label assigned based on our judgement.

Nr.	Category	Example	Gold label	Classifier label	Possible label	Remark
1	Easy	@USER Nigga ware da hits at	OFF	OFF	/	
2	Easy	#I once read about the man who invented bulbs, he saw failure differently, it was 5,000 new ways of doing it differently…Now he is a genius#	NOT	NOT	/	
3	Hard	#ChristineBlaseyFord is your #Kavanaugh accuser… #Liberals try this EVERY time… #ConfirmJudgeKavanaugh URL	OFF	NOT	/	Lack of context—from URL
	Hard	@USER @USER @USER Are you referring to how they lollygag with gun control as their kids get slaughtered in schools?	NOT	OFF	/	Lack of context
5	Ambiguous	#Kavanaugh so a wild claim from 36 years ago of groping has evolved into A Rape A Violent Sexual Event by Move URL a Soros based Org. that supports BLM Antifa etc. Unbelievable!	NOT	OFF	OFF	Gold label mistake. Offensive towards BLM etc.
6	Ambiguous	#Cancer Hate crime isn’t an actual crime. If there’s a victim, then it’s just called crime. (Malum in se) “Hate” crime is socially engineered programing to control people’s behavior, just like gun control. #ChangeMyMind URL*	NOT	OFF	BOTH	Both labels are possible. Giving opinion but could also be treated as pointing to blame to a gr. of people

**Table 8:** Slovenian examples from the test set belonging to different regions in the confidence-variability plot with gold labels and classifier labels. Examples which seem to have been assigned an incorrect gold label are given a possible correct label assigned based on our judgement.

Nr.	Category	Example	Gold Label	Classifier label	Possible Label	Remark
1	Easy	Postreljati to smrdljivo golazen pa direktno na grmado pa skurit [*Shoot this stinky vermin and burn it at the stake*]	OFF	OFF	/	
2	Easy	Been here, seen that :)	NOT	NOT	/	Different language
3	Hard	Na imigrantski mladini svet “stoji”. https://www.youtube.com/watch?v=Wj9LLC7GZQk Pridruži se, če ti ni vseeno za svojo domovino: https://www.facebook.com/stranka.slovenskega.naroda.ssn [*The world depends on young migrants. Join if you care about your country*.]	NOT	OFF	/	Lack of context—from URL
4	Hard	V zivalski vrt jh iskat pa bo zadeva resena :) [*Go to the zoo and get them, problem solved :)*]	NOT	OFF	/	Lack of context
5	Ambiguous	Sej bo ze drzava placala ne skrb haha [*Don’t worry, the government will pay haha*]	OFF	NOT	/	Lack of context
6	Ambiguous	Ce si rojen v sloveniji, to ne pomeni tud da si!!!!!!!!!!!!!vazne so korenine!!!!!!!! [*If you’re born in Slovenia it doesn’t mean you are a Slovenian!!!!!! Your roots matter!!!!!!*]	NOT	NOT	OFF	Gold label mistake

**Table 9:** Croatian examples from the test set belonging to different regions in the data map with gold labels and classifier labels. Examples which seem to have been assigned an incorrect gold label have a possible correct label assigned based on our judgment.

Nr.	Category	Example	Gold label	Classifier label	Possible label	Remark
	Easy	Ja san dobia zuti karton jer san covika oslovia sa klaune a to sto oni reklamiraju javno prostituciju, lazi, itd nikome nista… Admini ove stranice naguzite se mamicu [*I got a warning because I said to someone that he was a clown but they are advertising public prostitution, spreading lies etc. and nothing happens*… *Admins of this site are motherfuckers*.]	OFF	OFF	/	
2	Easy	Ko si ti kurvo glupa da nekome nešto govoris [*Who are you stupid whore to lecture someone*]	OFF	OFF	/	
3	Hard	Treba iz objesiti ! [*Needs to be hanged!*]	OFF	NOT	/	Lack of context
4	Hard	Gospođo, u kuhinju! [*Go to the kitchen, miss!*]	OFF	NOT	/	Sociolinguistic features
5	Ambiguous	Vaso jedi kurac [*Vaso eat dick*]	NOT	OFF	OFF	Gold label mistake
6	Ambiguous	Da je pravde po mom na ovom svijetu završile bi njemu ruke na giljotini pa nek boksa ćaću svog… Dizat ruku na Policiju ma mrs tamo [*If there were justice in this world his hands would end up on a guillotine and then he could start hitting his father… Striking a policeman, what the hell*]	NOT	OFF	OFF	Gold label mistake

For the Slovenian dataset, we found some examples written in a language other than Slovenian (see example 2 Table 8). We observe that on average such instances tend to get correctly classified, perhaps due to the effectiveness of the multilingual mBERT and cseBERT representations, or because the English used in these cases is relatively simple; however, no conclusions can be made without deeper analysis.

For Slovenian and Croatian, another category of examples was found that cannot be labeled without more general cultural and societal knowledge. We currently do not know how much such knowledge, if any, a language model possesses, which may lead to difficulties in labeling such messages. A clear-cut example would be “Gospodo, u kuhinju!” (Go to the kitchen, miss!) from the Croatian dataset (see Table 9). Such an example may seem very tame in terms of its vocabulary; however, in gender roles, it may be labeled as offensive to women. Such examples can be found in any region (easy, hard or ambiguous) of the data map. This suggests the classifier seems to pick some signals for these kinds of instances during training, however, the results are highly inconsistent. In order for the classifier to classify such instances correctly, it seems likely that similar instances must be present in the training set during fine-tuning; the knowledge from the pre-trained model may not be enough to decode such instances properly.

#### Attention visualization

**Figure 7:**
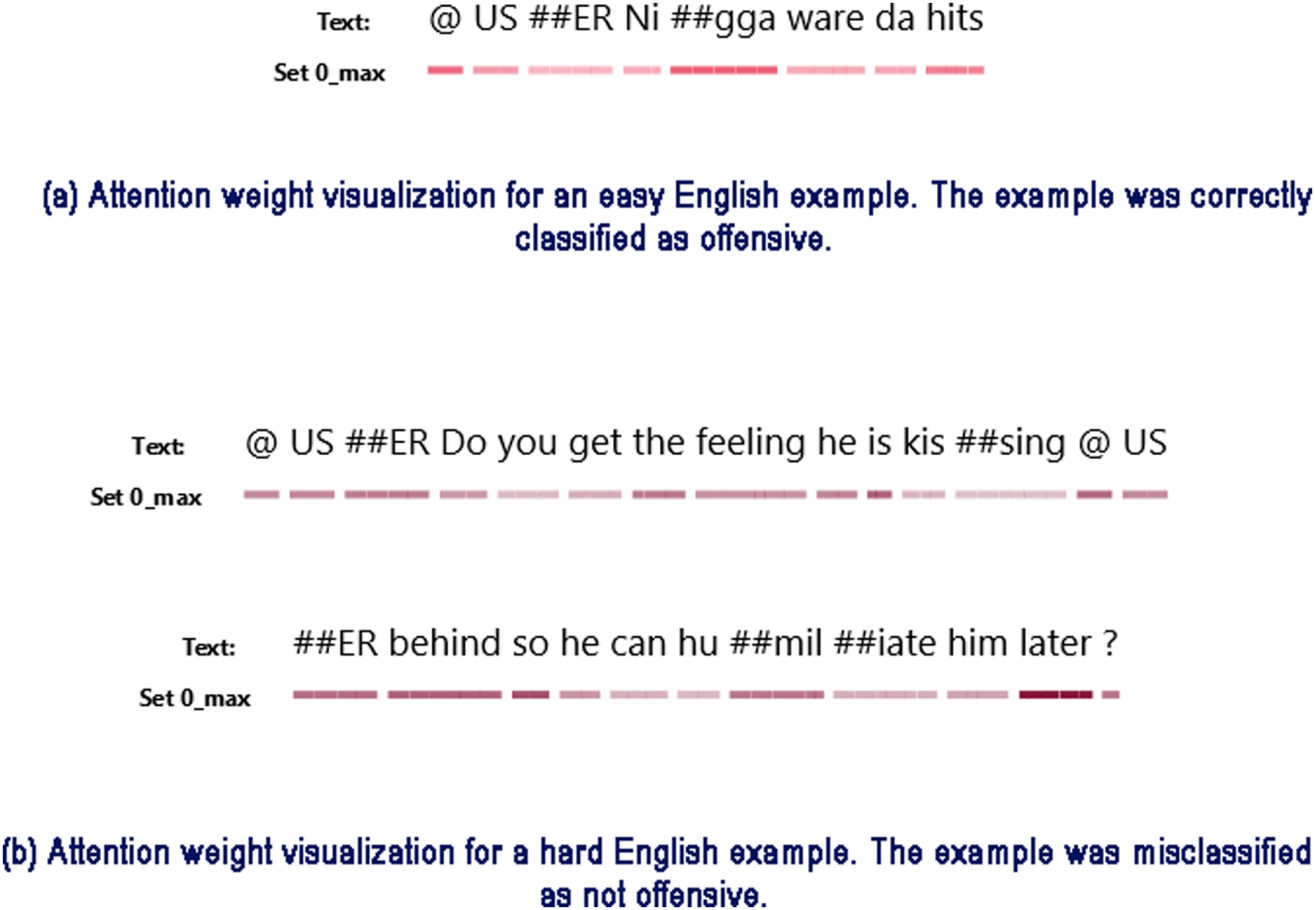
Attention weight comparison for an easy (A) and a hard (B) example in English.

In Fig. 7 we provide an attention weight visualization for two English examples, one from the high-confidence/low-variability region (i.e., “easy”) and another from the low-confidence/low-variability region of the data map (“hard”). For each instance we have visualized the maximum attention weight each token gets across BERT’s 12 attention heads, using the AttViz visualization tool (Škrlj et al., 2021). Since the role of attention is to weight different parts of the input, this lets us gauge the relative importance of specific input tokens.

As is standard with BERT models, we add two special tokens to the original input text during training and inference stages (see Devlin et al., 2019). The (CLS) token is added in the first position in the sequence, and its representation is used for performing classification. The (SEP) token is added in the last position of the input text sequence to mark its end. Since these two tokens are present in every input at predefined positions they are assigned high attention weights by the model. However, we are more interested in the importance of other tokens that are originally part of the input text. Since the presence of these two tokens during visualization may overshadow the importance of other tokens, we remove them from the input during visualization of the attention weights.

Figure 7A presents an “easy” example which was correctly classified by the model as offensive. We can see that the model puts a lot of weight on the token “##gga”, part of the offensive word “nigga”. It also puts moderate weight on the final word "hits" which may suggest violence. Figure 7B presents a “hard” English example. Here the model puts weight on the token “behind”, however it is unable to decipher the meaning of the English expression “kissing someone’s behind” and misclassifies the example as not offensive.

### Analysis of vocabulary coverage

In this section, we shed some light on the performance difference based on vocabulary coverage. Specifically, we are interested in understanding whether better vocabulary coverage helps classification performance. To measure this, we calculated the percentage of missing words in the sentence, i.e., the words that are not present either in the pre-trained LM vocabulary or in the training set. BERT-based models use WordPiece (Schuster & Nakajima, 2012; Wu et al., 2016) to create the vocabulary. WordPiece is a data-driven approach guaranteed to generate a deterministic segmentation of a word. For example, if “bagpipe” is not present in the vocabulary, but “bag” and “pipe” are, then “bagpipe” will be divided into two sub-words “bag” and “##pipe”, where “##” indicates that a token is part of the previous word. This allows for wider vocabulary coverage, as even rare words can be covered via their sub-word units. We define a missing word as either:

- a word *split to character level* (and therefore not in the pre-trained model’s vocabulary, although it may be present in the training data). The hypothesis behind this condition is that if words are split into individual characters rather than longer tokens, it is unlikely that a model can easily assign meaning.

or

- a word *not in the vocabulary nor in the training set*. In this case, a word may be split into larger units than characters. If the word is present in the training set, it is not considered as missing: the meaning may at least partly be learned by the classifier model during the training phase.

We illustrate this with an example sentence “I like flowers”, assuming that only “I” is present in the vocabulary, but “like” and “flowers” are present in the training set. If the sentence is tokenized as “I li ##ke flower ##s”, then there are 0 missing words. However, if tokenized as “I l ##i ##k ##e flower ##s” (i.e., “like” is character-level tokenized), there is one missing word, i.e., 33.33%.

**Figure 8:**
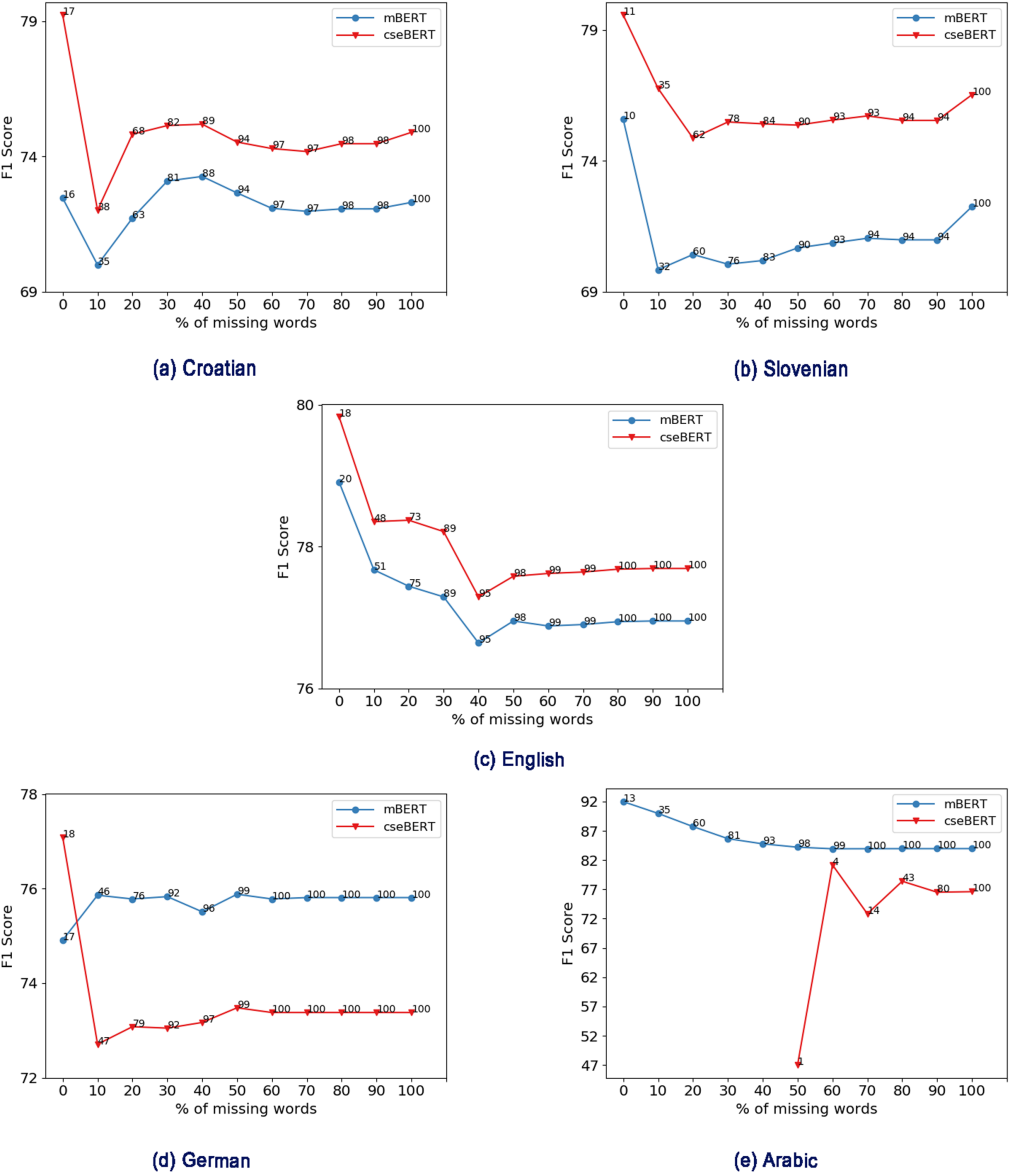
Effect of % of missing words (e.g., 30% means 30% or less missing words) on performance for mBERT and cseBERT. (A) Croatian, (B) Slovenian, (C) English, (D) German, (E) Arabic. Numbers on the lines represent % of test set samples covered at that point.

In Fig. 8, we plot the classifier F1 score against the cumulative percentage of missing words (i.e., for data with x% or less missing words, what is the performance). We also report the percentage of test set examples covered at that point. As we can see from Fig. 8, as the percentage of missing words increases, the performance decreases in most cases. There are a few exceptions: for Croatian, due to a sharp drop at 10% there is a large subsequent increase in performance. This could be due to more hard examples in that range.

For Croatian and Slovenian, cseBERT has fewer missing words than mBERT, and this better vocabulary coverage may be one reason for the performance gain. As we can see from Figures 8A and 8B, when there is less than 20% of missing words, cseBERT covers 3–5% more sentences for Croatian and Slovenian compared to mBERT, and shows a corresponding performance gain of more than 5–6%. However, this cannot be the only factor: at 0% missing words, even though there is only 1% higher dataset coverage, there is a large difference (4–5%) in performance. This could be due to larger whole-word vocabulary coverage, allowing cseBERT to learn better word meaning.

Interestingly for English (Fig. 8C), even though cseBERT has less vocabulary coverage, it performs slightly better. However, for German, the trend is the opposite: mBERT has less vocabulary coverage, and performs better, because it is pre-trained on the German data, while cseBERT is not. For Arabic, cseBERT has a very high percentage of missing words, with all the examples having more than 50% missing words (see Fig. 8E), and the difference between the cseBERT and mBERT performance is very high (11%, see Table 2).^4^ 4Please note that even though cseBERT is not trained on the Arabic script, it has some Arabic characters in the vocabulary and the Arabic dataset has some Latin words. Our results therefore show some links between vocabulary coverage and performance, but suggest that more research is needed to fully understand them. In the future, we plan to look at how these effects relate to word frequency and part of speech.

## Conclusion

In this work, we study the feasibility of cross-lingual training to develop offensive speech detection models. Specifically, we investigated how the choice of pre-trained multilingual language models and non-target language intermediate training impact the final performance. We experimented with five diverse languages; Croatian, Slovenian, English, German, and Arabic, using two pre-trained language models, mBERT and cseBERT. We found out that having a language model pre-trained with a smaller set of languages has a better overall performance than a general multilingual language model for those languages, and gives better performance via intermediate training. In general, intermediate training is not useful if a large amount of target language data is available, giving relatively small improvements in only approximately half of the experiments, regardless of choice of language or number of languages for intermediate training. However, intermediate training is useful when we have limited target language data, and is particularly effective with a good choice of pre-trained language model. In this case, intermediate training with all other available languages (LOO) boosted performance for all languages except German.

Considering the choice of language model had the most significant impact on the final model performance, we also performed a qualitative analysis of the two language models we used in this study, namely mBERT and cseBERT. Vocabulary analysis suggests that better vocabulary coverage could be one reason for better performance, but that it is probably not the only factor. The analysis using classifier confidence revealed that models generally have trouble classifying instances that are hard to understand without additional context. Furthermore, the models perform inconsistently where additional socio-political knowledge is required to label the message correctly.

In future work on cross-lingual hate speech detection, we would like to make our analysis more general by extending it to other languages and other NLP tasks, and extend our study to other multilingual language models beyond the BERT architecture, such as those based on XLM (Conneau & Lample, 2019).

## Appendix

We present additional metrics to better gauge the performance of our models in various experimental settings conducted in the course of this study.

Tables 10 and 11 show the results of mBERT and cseBERT models respectively in terms of macro-averaged recall and precision when they are trained on all available target language data without intermediate training. For comparison with the F1 score, refer to the Table 2.

**Table 10:** Results for mBERT models, fine-tuned on all training data in the target language only (no intermediate training). Values are shown as recall and precision scores with standard deviation. Bold indicates the best performance for each language.

Language	Recall		Precision	
	Random init.	Fine-tuned	Random init.	Fine-tuned
Croatian	51.68_1.9_	**69.14**_1.3_	51.56_1.8_	**74.70**_1.6_
Slovenian	49.30_19.0_	**72.67**_0.4_	47.82_3.7_	**72.87**_0.4_
English	51.70_1.9_	**75.89**_1.1_	52.14_1.6_	**77.56**_1.3_
German	49.47_0.5_	**75.16**_0.3_	48.33_0.4_	**77.14**_0.6_
Arabic	49.13_1.5_	**83.48**_0.6_	48.39_1.8_	**85.98**_0.4_

**Table 11:** Results for cseBERT models, fine-tuned on all training data in the target language only (no intermediate training). Values are shown as recall and precision scores with standard deviation. Bold indicates the best performance for each language.

Language	Recall		Precision	
	Random init.	Fine-tuned	Random init.	Fine-tuned
Croatian	48.34_2.2_	**73.38**_0.9_	48.77_1.6_	**77.33**_1.5_
Slovenian	48.96_1.6_	**76.17**_0.5_	49.15_1.9_	**76.11**_0.6_
English	50.91_1.2_	**76.46**_1.2_	50.87_0.9_	**77.88**_1.5_
German	50.90_2.4_	**73.38**_1.1_	56.70_7.9_	**74.96**_0.9_
Arabic	51.48_4.4_	**74.32**_0.9_	50.94_3.6_	**78.46**_1.2_

Tables 12 and 13 show the results of mBERT and cseBERT models respectively when intermediate training is performed in one or more non-target languages and no fine-tuning is performed on target language data (zero-shot setting). The performance of the models is measured in terms of macro-averaged recall and macro-averaged precision scores. For comparison with the F1 score, refer to Table 3.

**Table 12:** Results of intermediate training in a range of non-target languages in zero-shot transfer on the target language data for mBERT models using macro-averaged recall (top) and macro-averaged precision (bottom) scores. TGT: random initialization (no intermediate training, no target fine-tuning). ENG/SLO/AR → TGT: Intermediate training on English/Slovenian/Arabic, then zero-shot transfer on the target language. LOO → TGT: Intermediate training on all non-target languages, then zero-shot transfer on the target language. Bold indicates the best performance for each language.

Target	TGT	ENG → TGT	SLO → TGT	AR → TGT	LOO → TGT
	**Recall**				
Croatian	51.68_1.9_	↑55.66_0.0_	↑**65.96**_0.0_	↓50.44_0.0_	↑65.48_0.0_
Slovenian	49.30_19.0_	↑53.25_0.0_	–	↑51.69_0.0_	↑**56.16**_0.0_
English	51.70_1.9_	–	↓51.34_0.0_	↓50.73_0.0_	↑**54.21**_0.0_
German	**49.47**_0.5_	↓46.76_0.0_	↓45.76_0.0_	↓47.33_0.0_	↓41.70_0.0_
Arabic	49.13_1.5_	↑50.31_0.0_	↑**56.80**_0.0_	–	↑55.40_0.0_
	**Precision**				
Croatian	51.56_1.8_	↑**65.85**_0.0_	↑61.96_0.0_	↑51.76_0.0_	↑62.47_0.0_
Slovenian	47.82_3.7_	↑62.82_0.0_	–	↑64.51_0.5_	↑**65.70**_0.0_
English	52.14_1.6_	–	↑69.65_0.0_	↑52.31_0.0_	↑61.96_0.0_
German	**48.33**_0.4_	↓38.32_0.0_	↓39.83_0.0_	↓43.32_0.0_	↓32.93_0.0_
Arabic	49.93_1.8_	↑**89.85**_0.0_	↑64.14_0.0_	–	↑62.41_0.0_

**Table 13:** Results of intermediate training in a range of non-target languages in zero-shot transfer on the target language data for cseBERT models using macro-averaged recall (top) and macro-averaged precision (bottom) scores. TGT: random initialization (no intermediate training, no target fine-tuning). ENG/SLO/AR →TGT: Intermediate training on English/Slovenian/Arabic, then zero-shot transfer on the target language. LOO → TGT: Intermediate training on all non-target languages, then zero-shot transfer on the target language. Bold indicates the best performance for each language.

Target	TGT	ENG → TGT	SLO → TGT	AR → TGT	LOO → TGT
	**Recall**				
Croatian	48.34_2.2_	↑**72.87**_0.0_	↑70.19_0.0_	↑49.51_0.0_	↑71.97_0.0_
Slovenian	48.96_1.6_	↑**66.81**_0.0_	–	↓49.79_0.0_	↑60.13_0.0_
English	50.91_1.2_	–	↑58.13_0.0_	↓49.84_0.0_	↑**61.26**_0.0_
German	**50.90**_2.4_	↓49.38_0.0_	↓50.11_0.0_	↓50.54_0.0_	↓50.10_0.0_
Arabic	**51.48**_4.4_	↓50.31_0.0_	↓50.31_0.0_	–	↓50.63_0.0_
	**Precision**				
Croatian	48.77_1.6_	↑**67.63**_0.0_	↑67.34_0.0_	↓38.75_0.0_	↑66.62_0.0_
Slovenian	49.15_1.9_	↑**69.52**_0.0_	–	↑45.45_0.0_	↑68.22_0.0_
English	50.87_0.9_	–	↑73.75_0.0_	↓36.01_0.0_	↑**77.15**_0.0_
German	56.70_7.9_	↓36.02_0.0_	↓54.94_0.0_	↓55.77_0.0_	↑**67.43**_0.0_
Arabic	50.94_3.6_	↑89.85_0.0_	↑89.85_0.0_	–	↑**89.90**_0.0_

Tables 14 and 15 show the results of mBERT and cseBERT models respectively when intermediate training is performed in one or more non-target languages and fine-tuning is performed on all available target language data. The performance of the models is measured in terms of macro-averaged recall and macro-averaged precision scores. For comparison with the F1 score, refer to Table 4.

**Table 14:** Results of intermediate training in a range of non-target languages, followed by fine-tuning on all target language data for mBERT models using macro-averaged recall (top) and macro-averaged precision (bottom) scores. TGT: Only fine-tuned on target language (no intermediate training). ENG/SLO/AR → TGT: Intermediate training on English/Slovenian/Arabic, then fine-tuning on target language. LOO → TGT: Intermediate training on all non-target languages, then fine-tuning on target language. Bold indicates the best performance for each language.

Target	TGT	ENG → TGT	SLO → TGT	AR → TGT	LOO → TGT
	**Recall**				
Croatian	69.14_1.3_	↑70.06_1.6_	↑**70.14**_0.4_	↑69.92_0.8_	↑69.57_0.3_
Slovenian	72.67_0.4_	↓72.26_1.1_	–	↑73.83_0.7_	↑**74.95**_1.0_
English	**75.89**_1.1_	–	↓73.18_0.6_	↓73.92_0.6_	↓75.25_0.6_
German	75.16_0.3_	↑**75.25**_0.2_	↓73.89_0.1_	↓74.21_1.2_	↓74.23_0.6_
Arabic	83.48_0.6_	↓82.83_1.1_	↑84.55_1.3_	–	↑**84.06**_0.6_
	**Precision**				
Croatian	74.70_1.6_	↑75.35_1.6_	↑**75.58**_0.8_	↑75.41_1.4_	↑74.85_1.5_
Slovenian	72.87_0.4_	↓72.75_0.9_	–	↑74.03_0.6_	↑**75.10**_1.2_
English	**77.56**_1.3_	–	↓75.33_1.8_	↓75.83_0.3_	↓77.20_0.9_
German	77.14_0.6_	↑**77.46**_0.4_	↓75.30_0.0_	↓76.06_1.0_	↓76.40_0.2_
Arabic	85.98_0.4_	↓85.61_0.5_	↑87.16_0.6_	–	↑**87.37**_0.5_

**Table 15:** Results of intermediate training in a range of non-target languages, followed by fine-tuning on all target language data for cseBERT models using macro-averaged recall (top) and macro-averaged precision (bottom) scores. TGT: Only fine-tuned on target language (no intermediate training). ENG/SLO/AR → TGT: Intermediate training on English/Slovenian/Arabic, then fine-tuning on target language. LOO → TGT: Intermediate training on all non-target languages, then fine-tuning on target language. Bold indicates the best performance for each language.

Target	TGT	ENG → TGT	SLO → TGT	AR → TGT	LOO → TGT
	**Recall**				
Croatian	73.38_0.9_	↑74.66_1.2_	↓73.35_0.5_	↓73.21_0.5_	↑**74.67**_0.8_
Slovenian	76.17_0.5_	↑**76.76**_0.4_	–	↓76.10_0.5_	↑76.48_0.3_
English	76.46_1.2_	–	↓76.25_0.8_	↓76.17_1.2_	↑**76.70**_0.5_
German	**73.38**_1.1_	↓70.85_1.1_	↓68.37_0.4_	↓69.88_0.3_	↓68.69_0.8_
Arabic	74.32_0.9_	↑75.09_0.5_	↑74.89_1.3_	–	↑**76.72**_1.4_
	**Precision**				
Croatian	77.33_1.5_	↑**79.41**_0.9_	↓77.26_1.0_	↑78.93_1.1_	↑77.80_0.4_
Slovenian	76.11_0.6_	↑**76.83**_0.3_	–	↓76.05_0.5_	↑76.40_0.3_
English	77.88_1.5_	–	↑78.26_1.0_	↑78.25_0.6_	↑**79.11**_0.1_
German	**74.96**_0.9_	↓73.10_0.8_	↓72.10_0.5_	↓71.66_0.2_	↓70.67_1.0_
Arabic	78.46_1.2_	↓78.18_0.7_	↑78.97_2.0_	–	↑**81.08**_1.6_

The additional metrics seem to confirm our claims of model comparison between mBERT and cseBERT models. Both in scenarios where high amounts of target language data are available and in scenarios where target language data is not available (zero-shot scenario), the cseBERT consistently shows higher performance than mBERT on Croatian, Slovenian and English languages.

## Supplemental Information

10.7717/peerj-cs.559/supp-1 Supplemental Information 1 Slovenian FRENK dataset splits. **Review Only**
The dataset splits that were used in the experiment for the Slovenian language

## Additional Information and Declarations

### Data Availability

The following information was supplied regarding data availability: The SemEval corpora in English, German and Arabic datasets are available under the Creative Commons Attribution 4.0 International License, and the training/evaluation/test splits for exact reproduction of our experiments are available at: https://github.com/EMBEDDIA/cross-lingual_training_for_offensive_language_detection. The Slovenian data splits that we used in our experiments were provided for peer-review; the code used to split the Slovenian dataset is available at GitHub (in the module data_prep.py): https://github.com/EMBEDDIA/cross-lingual_training_for_offensive_language_detection. For Croatian (24sata), the data is available as part of the EMBEDDIA project and is available at: https://www.clarin.si/repository/xmlui/handle/11356/1399.
